# A general protocol for engineering metal–oxo-chain standing frameworks

**DOI:** 10.1093/nsr/nwag018

**Published:** 2026-01-15

**Authors:** Jun Guo, Zhiyong Ban, Yutian Qin, Siyang Li, Zelong Zhao, Yongli Ji, Peter E VanNatta, Yin Zhang, Meiting Zhao, Thamraa AlShahrani, Shengqian Ma

**Affiliations:** State Key Laboratory of Advanced Separation Membrane Materials, School of Electronics and Information Engineering & School of Chemistry, Tiangong University, Tianjin 300387, China; State Key Laboratory of Advanced Separation Membrane Materials, School of Electronics and Information Engineering & School of Chemistry, Tiangong University, Tianjin 300387, China; Tianjin Key Laboratory of Molecular Optoelectronic Sciences, Department of Chemistry, Institute of Molecular Aggregation Science, Tianjin University, Tianjin 300072, China; CAS Key Laboratory of Nanosystem and Hierarchical Fabrication, CAS Center for Excellence in Nanoscience, National Center for Nanoscience and Technology, Beijing 100190, China; State Key Laboratory of Advanced Separation Membrane Materials, School of Electronics and Information Engineering & School of Chemistry, Tiangong University, Tianjin 300387, China; State Key Laboratory of Advanced Separation Membrane Materials, School of Electronics and Information Engineering & School of Chemistry, Tiangong University, Tianjin 300387, China; Department of Chemistry, University of North Texas, Denton, TX 76201, USA; Department of Chemistry, University of North Texas, Denton, TX 76201, USA; Tianjin Key Laboratory of Molecular Optoelectronic Sciences, Department of Chemistry, Institute of Molecular Aggregation Science, Tianjin University, Tianjin 300072, China; Department of Physics, College of Science, Princess Nourah bint Abdulrahman University, Riyadh 11564, Saudi Arabia; Department of Chemistry, University of North Texas, Denton, TX 76201, USA

**Keywords:** metal–oxo, infinite secondary structure, metal-organic framework, hydrodeoxygenation

## Abstract

Infinite metal–oxo metal–organic frameworks (MOFs) are recognized as promising platforms for developing all-round high-performance catalysts for both academic and industrial significance. Nevertheless, engineering infinite metal–oxo architectures typically requires harsh synthetic conditions, often yielding microcrystalline or even nanocrystalline products that hinder precise structure identifications. Herein, we propose a previously underestimated acetic acid-based solvothermal protocol for general engineering of 1D infinite metal–oxo (e.g. Zr, Hf, Ce) MOFs featuring large-size single crystals with well-identified crystallographic structures. As an example, the 1D Zr–BTB-derived catalyst exhibits a turnover frequency (TOF) of 1199.1 h^−1^, selectivity of 99.0% and long-term stability in the catalytic upgrading of natural feedstocks into high-value-added fuels. In comparison, the conventional Zr_6_O_8_ node-based counterpart only presents a TOF of 282.5 h^−1^, selectivity of 5.9% and poor recycling ability. This work opens the avenue to design industry-oriented performant heterogeneous catalysts for energy-critical transformations via rational engineering of versatile infinite metal–oxo units.

## Teaser

An unusual acetic acid-based synthetic protocol is proposed for generally engineering single-crystal metal-oxo-chain standing frameworks competent for industry-oriented performant heterogeneous catalysts with all-excellence in activity, selectivity, and durability.

## INTRODUCTION

High catalytic activity, specific product selectivity and durable performance stability constitute cardinal criteria for evaluating high-performance catalysts in both academic research and industrial applications. Nevertheless, the irreconcilable trade-off between activity and stability renders the simultaneous realization of merely two superiorities within a single catalyst a long-standing challenge [1,2]. Among heterogeneous catalysts, for instance, enhanced surface area and more open structure are beneficial for improving catalytic activity by facilitating substrate diffusion and energy transfer. These same features, however, inherently elevate surface energy and hence make catalysts more prone to particle aggregation, porosity collapse and even structure decomposition under operational conditions [3–5].

Metal–organic frameworks (MOFs), as a class of crystalline porous materials, are well known for their ultrahigh surface areas, tunable pore structures, easy functionalization and well-defined crystallography [6–9]. Over recent decades, MOFs have achieved remarkable progress in catalyzing diverse organic transformations [10–12]. In comparison with conventional MOFs composed of discrete and untenable metal nodes [13,14], emerging MOFs assembled from robust metal–oxo infinite units such as 1D metal–oxo chains or 2D metal–oxo sheets represent a unique subclass exhibiting exceptional physicochemical properties and remarkable stabilities [15–17]. For instance, they can exhibit ultrahigh thermal [18,19], hydrolytic [20] and mechanical [21,22] stabilities, meanwhile exposing abundant highly active metal–oxo sites that are particularly suited for catalytic applications [19,23–25]. As a result, infinite metal–oxo MOFs have emerged as promising candidates for achieving double or even multiple goals including superior catalytic activity [26], product selectivity and durable stability. However, synthesizing such infinite metal–oxo structures in MOFs, as opposed to engineering discrete metal nodes, typically requires harsh synthesis conditions such as elevated reaction temperatures, boosted pressures and concentrated precursors [18]. Unfortunately, such conditions often excessively accelerate reaction kinetics and therefore yield microcrystalline or even nanocrystalline MOF powders, which are incompatible with single-crystal X-ray diffraction (SXRD) identifications. This limitation has resulted in scarce precision in structural characterizations, with many reported examples relying on computer-predicted models or/and indirect powder diffraction refinements [17–19,23–25,27–29].

**Figure 1.**
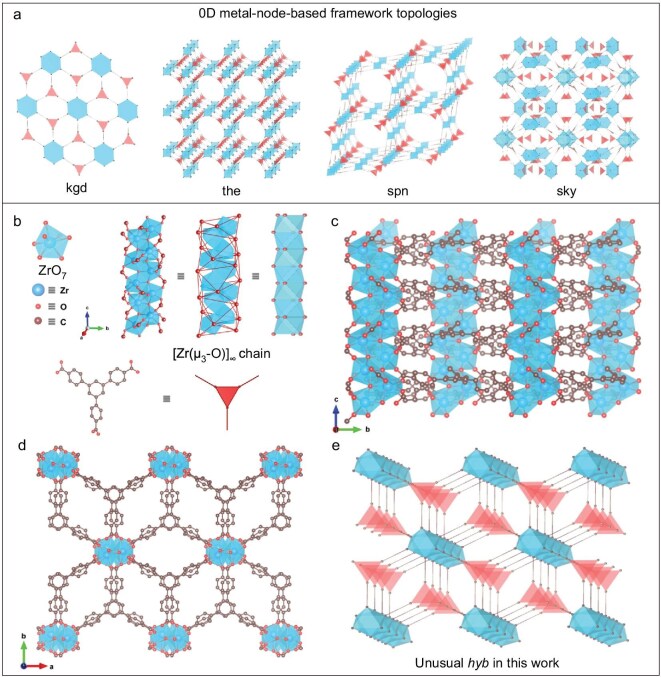
SXRD characterization and topology analysis of the Zr–BTB phase. (a) Conventional framework topologies assembled from discrete Zr_6_O_8_ node and tridentate linker. (b) Zr–BTB framework comprises 1D zigzag [Zr(μ_3_-O)]_∞_ chains and 3-connected BTB ligands. The [Zr(μ_3_-O)]_∞_ chain is composed of ZrO_7_ polyhedra and bridged μ_3_-O ions. The [Zr(μ_3_-O)]_∞_ chain and BTB linker can be abstracted as a 1D face-sharing octahedral pillar and an augmented 3-connected triangle, respectively. (c and d) The crystal structure of Zr–BTB along the projection of the *a*-axis and *c*-axis, respectively. (e) Zr–BTB presents an unusual binodal (3,7)-connected *hyb* topology with the vertex symbol of {3⋅8^2^}{3^7^⋅4^6^⋅5^2^⋅8^4^⋅9^2^}.

In this contribution, we present an unconventional acetic acid-based solvothermal synthesis protocol for general engineering of 1D metal–oxo MOFs. Distinct from previous approaches using monoacids like acetic acid solely as modulators [18,23,24], we utilize it directly as the reaction solvent for several compelling reasons. Firstly, acetic acid effectively suppresses the hydrolysis of metal precursors into conventional discrete metal nodes, thermodynamically facilitating formation of unusual metal–oxo MOFs with high phase purity. Secondly, limited dissolvability of organic linkers in acetic acid slows both nucleation and growth processes of MOFs, even at ultrahigh reaction temperatures and pressures, enabling cultivation of large MOF single crystals suitable for further SXRD characterization. Finally, acetic acid concurrently acts as a coordination modulator competing with carboxylic linkers and therefore confers abundant catalytically active sites for further applications. As a proof of concept, we successfully synthesized novel Zr–BTB [Zr_3_(μ_3_-O)_3_(BTB)_2_; BTB = 1,3,5-tris(4-carboxyphenyl)benzene] MOF occurring as large single crystals. SXRD analysis reveals its unique structure composed of zigzag 1D [Zr(μ_3_-O)]_∞_ chains interconnected by a tritopic BTB linker in an unusual binodal (3,7)-connected *hyb* topology differing from conventional Zr_6_O_8_-based frameworks (Fig. 1a) [30–32]. Crucially, this acetic acid-based solvothermal protocol demonstrates broad applicability, enabling synthesis of metal–oxo MOFs with alternative metal types (e.g. Hf and Ce), as well as diverse organic linkers (e.g. bidentate 1,4-benzenedicarboxylate, tritopic BTB and tetradentate porphyrin derivative).

Furthermore, the catalytic performance of Zr–BTB MOF after supporting Pd nanoparticles (NPs) was assessed in the selective hydrodeoxygenation of lignin-derived feedstocks, a crucial process for upgrading natural biomass into non-renewable fossil resources [33] while demanding robust catalysts withstanding harsh operation conditions [34]. To highlight the structurally derived advantages, we also compared performance in parallel against the UMCM-309 polymorph [Zr_6_(μ_3_-O)_4_(μ_3_-OH)_4_(BTB)_2_(OH)_6_] [31]. Benefitting from higher accessibility and stronger Lewis acidity inherent to 1D [Zr(μ_3_-O)]_∞_ chains, Pd@Zr–BTB achieved a conversion rate of 99.9% within only 0.75 h, a turnover frequency (TOF) of 1199.1 h^−1^, as well as 99.0% specific selectivity toward the production of high-caloric *p*-creosol fuel. In striking contrast, the counterpart Pd@UMCM-309 exhibited markedly inferior performance of only 282.5 h^−1^ TOF and 5.9% *p*-creosol selectivity. The high performances (99.0% conversion rate and >95.0% selectivity) acquired by Pd@Zr–BTB are further extended to 10 kinds of vanillin-like substrates. Furthermore, structurally robust Zr–oxo chains endowed Zr–BTB with exceptional recyclability, maintaining undecayed catalytic activity, as well as product selectivity, even over 10 consecutive catalysis cycles.

## RESULTS AND DISCUSSION

Zr–BTB single crystals (Fig. S1) with morphological parameters of ∼95.0 μm length and ∼27.5 μm diameter (Fig. S2) are produced in acetic acid under the reaction temperature of 220°C (see the details in the Methods section of the Supplementary data). In contrast, conventional solvothermal synthesis in *N,N*-dimethylformamide (DMF) yielded only amorphous spherical particles (Fig. S7), underscoring acetic acid’s indispensable function in crystallizing Zr–BTB. Distinct from previously reported Zr–oxo MOF powders that were unexceptionally characterized by indirect powder X-ray diffraction (PXRD) refinement and computer modeling (Table S1), the absolute crystallographic structure of Zr–BTB has been unambiguously resolved by direct SXRD characterization thanks to its high single-crystal quality cultivated in acetic acid. The crystallographic information of Zr–BTB is summarized in Tables S2–S8 with finely converged indices (i.e. the final *R*_1_ = 0.0321, *wR*_2_ = 0.0793 and the goodness-of-fit value = 1.045). The corresponding crystallography information file (CIF) including all structure parameters is available from Cambridge Crystallographic Data Centre (CCDC) with an identifier number of 2040243 and is summarized in the Supplementary data. Specifically, Zr–BTB has the formula Zr_3_(μ_3_-O)_3_(BTB)_2_ and crystallizes in a monoclinic crystallographic system with the space group of *C*2/*c*. Unlike the 8-coordination mode in the conventional [Zr_6_(μ_3_-O)_4_(μ_3_-OH)_4_] node [14], Zr(IV) ions in Zr–BTB adopt an unusual 7-connected coordination with four mono-coordinated carboxylate oxygens and three μ_3_-bridged oxygen (μ_3_-O) ions (Fig. 1b). Through Zr–(μ_3_-O) bridging bonds, a 1D infinite zigzag [Zr(μ_3_-O)]_∞_ chain is consequently formed along the *c*-axis and is further connected by the tritopic BTB ligand in the crystallographic *a–b* plane to form the final 3D network (Fig. 1c and d). To identify the underlying topology of Zr–BTB, the 1D Zr–oxo chain is abstracted by using carbon atoms of coordinated carboxylate as extension points according to the established topology decomposition principle [15,35,36]. As a result, each extension point adopts a 7-connected mode and encircles an octahedron, which further packs into an infinite 1D column along the *c*-axis via sharing polyhedral faces. Meanwhile, the 3-connected BTB linker spans the aforementioned 1D columns within the *a–b* plane. Finally, a binodal (3,7)-connected *hyb* topology (Fig. 1e) with the vertex symbol of {3⋅8^2^}{3^7^⋅4^6^⋅5^2^⋅8^4^⋅9^2^} is assigned to obtain the Zr–BTB phase according to the definition of the Reticular Chemistry Structure Resource (RCSR) [37]. Note that such *hyb* topology has still not appeared in the versatile Zr-based MOF family to our knowledge (Table S9).

**Figure 2.**
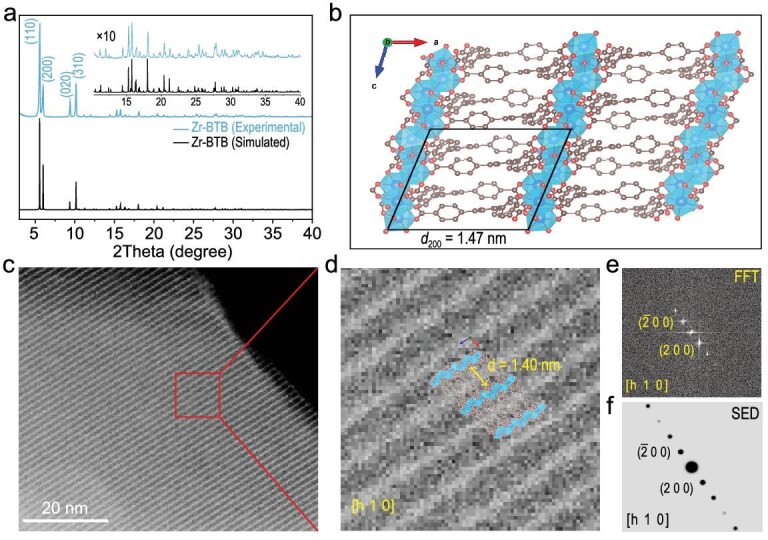
PXRD and TEM characterizations of Zr–BTB powders. (a) Experimental PXRD and calculated pattern based on SXRD. Inset shows the magnified region from 10.3° to 40°, highlighting the exceptional consistency between experimental and calculated results. (b) Crystal structure illustration of Zr–BTB viewed along the *b*-axis. The black rhomboid indicates a single lattice unit of Zr–BTB in the *a–c* plane with a corresponding *d*_200_ plane of 1.47 nm. (c) HR-TEM characterizations of Zr–BTB. The 1D lattice fringe is assigned to the Zr–oxo chain. (d) Magnification of the red solid rectangle region indicated in (c). (e) Derived FFT pattern of the lattice profile in (d). (f) Corresponding SED pattern based on SXRD.

Zr–BTB powders are subsequently attained for further characterizations and applications by simply increasing the precursor concentrations (see details in the Methods section of the Supplementary data). To confirm the phase purity of obtained Zr–BTB powders, PXRD was carried out and the collected pattern is rigorously compared with the calculated one based on SXRD. Zr–BTB powders (Fig. 2a) present a virtually identical diffraction pattern (blue plot) to the calculated one (black plot), and the four primary peaks centered at the 2*θ* values of 5.49°, 5.92°, 9.31° and 10.08° have been indexed to (110), (200), (020) and (310) planes, respectively. Instead of the invisible crystallinity of conventional MOFs upon exposure to highly energetic electron beams [38], 1D Zr–oxo chains in Zr–BTB frameworks are able to be observed by spherical aberration-corrected transmission electron microscope (SA-TEM) in the real space. In good accordance with the crystallographic structure (Fig. 2b), one can clearly see that 1D lattice fringes arrange in a parallel fashion within the Zr–BTB framework from both high-angle annular dark-field scanning (HAADF-STEM, Fig. 2c) and normal light-field TEM images (Fig. S3). The measured average lattice space is 1.40 nm (Fig. 2d) between parallel 1D Zr–oxo chains and assigned to the facet space of the (200) plane (1.47 nm) of Zr–BTB. Moreover, the fast Fourier transform (FFT) was further executed, producing a lattice pattern (Fig. 2e) consistent with the simulated electron diffractions (SEDs; Fig. 2f). The slightly shrunken lattice space relative to the crystallographic space of *d*_(200)_ may be attributed to the ultrahigh vacuum conditions during TEM characterizations [39].

Serving as a universal protocol, our synthetic strategy is also feasible for constructing diverse 1D metal–oxo MOFs of other metal types and organic ligands. For instance, isoreticular Hf–BTB single crystals with the same rod-like morphology (∼115.0 μm length and ∼42.5 μm diameter) have been successfully obtained using acetic acid-mediated solvothermal synthesis (Figs S8 and S9). Owing to the conserved high crystal quality (Figs S10 and S11), the crystallographic structure of Hf–BTB (CCDC No. 2330779) has also been comprehensively characterized by SXRD (Tables S10–S16), revealing nearly identical crystallographic parameters to those of Zr–BTB and confirming their isoreticular nature. Notably, Hf-MOFs [40] are typically more prone to be attained as nanosized powders via conventional solvothermal syntheses, even using excess modulators, due to its stronger hydrolysis ability than Zr(IV) one [41]. The success of Hf–BTB single crystals hence highlights the distinct advantage of acetic acid-mediated solvothermal protocol proposed in this work. Furthermore, the 1D metal–oxo secondary structure was also observed for the Ce analog, as demonstrated by the successful synthesis of the isoreticular Ce–BTB MOF (Figs S15–S17). Regarding the versatility towards organic ligands, bidentate tetrafluoroterephthalic acid (Figs S18–S20) and 4-connected tetrakis(4-carboxyphenyl) porphyrin ligands (Figs S21 and S22) have been successfully incorporated to construct corresponding metal–oxo-chain MOFs via the acetic acid-based solvothermal protocol. Furthermore, the protocol demonstrates broad applicability across diverse organic acids. For example, (Zr, Hf)–BTB MOFs were successfully synthesized using propionic acid (PA) or butyric acid (BA) instead of acetic acid under otherwise identical conditions (Figs S5, S6, S12 and S13). In summary, the demonstrated good applicability underscores our protocol’s great potential for developing novel MOFs featuring robust 1D metal–oxo chains.

**Figure 3.**
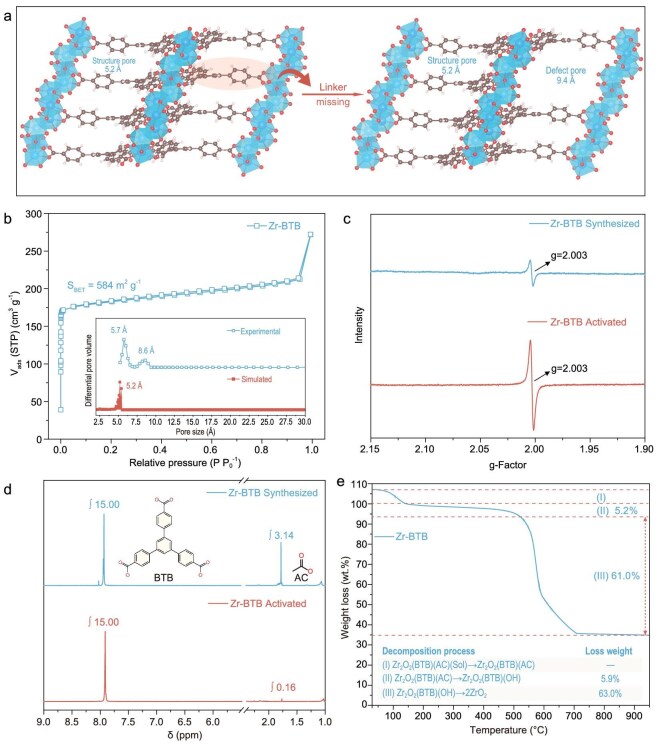
Defect structures in Zr–BTB via the missing BTB linker. (a) Schematic illustrating additional defect pores and open metal sites in Zr–BTB via the missing BTB linker. (b) N_2_ adsorption–desorption isotherms of Zr–BTB probed at 77 K. Inset shows the pore size distribution. (c) EPR spectra of as-synthesized Zr–BTB and activated Zr–BTB at 298 K. (d) ^1^H-NMR spectra of as-synthesized Zr–BTB and activated Zr–BTB via D_2_SO_4_-assisted dissolution in DMSO-d_6_. (e) TGA curve of Zr–BTB sample under N_2_ atmosphere.

Thanks to competitive coordination by acetic acid [40], additional pore defects are anticipated in 1D metal–oxo MOFs resulting from partially missed linkers (Fig. 3a). Taking Zr–BTB as the representative (Fig. 3b), its nitrogen (N_2_) sorption–desorption isotherms clearly show a typical type I isotherm characteristic of microporosity. Notably, the sharp N_2_ uptake near saturation pressure might arise from capillary condensation in interparticle voids formed by stacking rod-shaped crystallites. The corresponding Brunauer–Emmett–Teller (BET) surface area is calculated to be 584 m^2^/g (Fig. S23). Based on the non-local density functional theory (NLDFT), the experimentally measured pore size distribution of Zr–BTB (inset in Fig. 3b) shows a primary peak centered at 5.7 Å, which is in good agreement with the triangle channel (5.2 Å) simulated from the crystallographic structure model [42]. Notably, an additional peak centered at 8.6 Å is obviously discerned for the Zr–BTB sample, indicating the existence of defect porosity arising from the BTB linker being missing (Fig. 3a). According to the previously established protocol [43,44], missing linker defects of MOFs can also be detected through electron paramagnetic resonance (EPR) spectroscopy. As shown in Fig. 3c, a symmetrical differential signal at the proportionality factor (*g*-factor) of 2.003 is observed for Zr–BTB, which is further strengthened in intensity after activation, confirming the existence of abundant defects induced by missing linkers. In order to quantitatively characterize missing BTB linkers, proton nuclear magnetic resonance (^1^H-NMR) was performed on D_2_SO_4_-digested Zr–BTB samples before and after activation. As expected, the acetate hydrogens with the chemical shift of ∼1.75 ppm are clearly discerned for freshly synthesized Zr–BTB (Fig. 3d). On the basis of integrations of the peak areas, corresponding molar ratios of substituted acetate to BTB linker are quantitatively calculated to be nearly 1:1, corresponding to a formula of Zr_3_(μ_3_-O)_3_BTB_1.5_(OAc)_1.5_. Considering the lability of monocarboxylic modulators, metal sites occupied by acetate can be reopened as Lewis acid sites for further catalysis applications. As shown in Fig. 3d, acetate species in Zr–BTB are completely removed according to the negligible acetate ^1^H-NMR signals after the activation treatment.

Thermogravimetric analysis (TGA) was carried out to confirm the occupation of acetates, as well as characterize the thermal stabilities of the Zr–BTB framework armed with robust 1D [Zr(μ_3_-O)]_∞_ chains. As shown in Fig. 3e, initial weight loss below heating to ∼130°C is attributed to the evaporation of guest solvent molecules. A sequential weight loss of 5.2% is ascribed to gradual dissociation of substituted acetates, agreeing well with the theoretical value of 5.9% according to the estimated formula of Zr_3_(μ_3_-O)_3_BTB_1.5_(OAc)_1.5_ from the ^1^H-NMR result. Remarkably, Zr–BTB maintains excellent thermal stability with an onset decomposition temperature (*T*_d_) of 550°C despite significant linker deficiencies. The isoreticular Hf–BTB analog exhibits similarly ultrahigh stability (*T*_d_ = 542°C). Notably, these values surpass nearly all reported Zr/Hf–MOFs (Table S18). Bearing these in mind, the abundant open metal sites, straightforward channel structures and exceptional stability advance Zr–BTB as a promising candidate for further industry-oriented catalysis explorations.

The selective hydrodeoxygenation of lignin-derived vanillin to high-caloric liquid fuels represents a sustainable route to upgrade natural feedstocks into biofuels alternative to fossil resources [33,45,46]. Acting as a sequential reaction, vanillin is first hydrogenated to form vanillyl alcohol. However, its further transformation into the high-caloric *p*-creosol product is more difficult via a second hydrogenolysis step [46]. As a result, highly selective hydrodeoxygenation of vanillin to target *p*-creosol typically requires elevated reaction temperatures and high hydrogenation pressure [34]. Aside from sustainability and economic consideration, the applied harsh catalysis conditions also undermine the stability of employed catalysts, which is still formidably challenging among traditional MOFs [47,48]. By virtue of its straightforward pore channels facilitating mass and energy transport, abundant open metal sites serving as Lewis acid sites, and robust structural stability required for long-term operation, the activated 1D metal–oxo-chain framework Zr–BTB was utilized to support Pd NPs for vanillin hydrodeoxygenation. To underscore the catalytic advantages of the 1D metal–oxo structure, a conventional UMCM-309 MOF consisting of the same BTB linker but with discrete 0D Zr_6_(μ_3_-O)_4_ nodes has also been synthesized and activated for parallel evaluation (Fig. S24 and Figs S26–S28).

**Figure 4.**
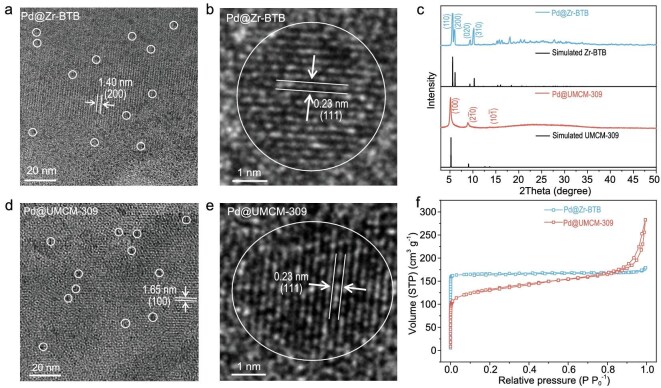
Characterizations of Pd@Zr–BTB and Pd@UMCM-309 catalysts. (a) HR-TEM image of Pd@Zr–BTB. The fringe is attributed to the *d*_(200)_ lattice of Zr–BTB. Pd NPs are highlighted with white circles. (b) HR-TEM image of Pd NPs in Pd@Zr–BTB. The fringe is attributed to the Pd *d*_(111)_ plane. (c) PXRD patterns of Pd@Zr–BTB and Pd@UMCM-309 catalysts. (d) HR-TEM image of Pd@UMCM-309. The fringe is attributed to the *d*_(100)_ lattice of UMCM-309. Pd NPs are highlighted with white circles. (e) HR-TEM image of Pd NP in Pd@UMCM-309. The fringe is attributed to the Pd *d*_(111)_ plane. (f) N_2_ adsorption–desorption isotherms of Pd@Zr–BTB and Pd@UMCM-309 catalysts probed at 77 K.

As shown in Fig. 4, tiny Pd NPs (∼3.5 nm) have been successfully incorporated into both Zr–BTB and UMCM-309 frameworks via double-solvent impregnation followed by sequential H_2_/Ar flow (10:90, v:v) treatment at 200°C. The resulting Pd@Zr–BTB and Pd@UMCM-309 maintain framework crystallinities, as evidenced by well-defined lattice fringes (Fig. 4a and d) and unchanged PXRD patterns (Fig. 4c) compared with raw Zr–BTB and UMCM-309, respectively. Both Pd@Zr–BTB and Pd@UMCM-309 exhibit uniformly distributed Pd nanoparticles with an average size of 3.5 nm (Figs S4 and S25). Note that the absence of characteristic diffraction peaks of Pd NPs shall be attributed to ultralow loading amounts of Pd NPs for Pd@Zr–BTB (0.82 wt%) and Pd@UMCM-309 (0.90 wt%) based on inductively coupled plasma-mass spectrometry (ICP-MS) results. The crystallinities of Pd NPs in both catalysts are demonstrated by their discernible *d*_(111)_ lattice of 0.23 nm from HR-TEM images (Fig. 4b and e). Moreover, N_2_ sorption tests confirm reserved pore structures of Pd@Zr–BTB and Pd@UMCM-309 (Fig. 4f) despite slightly reduced BET surface areas in comparison to pristine MOFs (Figs S29 and S30).

The catalytic hydrodeoxygenation of vanillin was conducted at 60°C and 0.5 MPa hydrogen pressure, considerably milder conditions than most reported studies (Table S19). As summarized in Table 1, the counterpart Pd@UMCM-309 gives an inferior vanillin conversion rate (44.2%), failing to produce the desired *p*-creosol, with a poor selectivity of 5.9%, while primarily yielding the hydrogenated vanillyl alcohol byproduct, with selectivity of 78.5%. In sharp contrast, Pd@Zr–BTB exhibits a remarkably high conversion ratio of vanillin (>99.9%) and, more importantly, specific selectivity of 99.0% towards target *p*-creosol within only 0.75 h (Entry 2, Table 1). Its kinetic TOF number calculated at a low conversion rate is as high as 1199.1 h^−1^. Similarly, Pd@Hf–BTB featuring abundant Hf–oxo sites exhibits >99.9% conversion rate and 97.9% *p*-creosol selectivity, with a kinetic TOF of 1044.9 h^−1^ (Entry 3, Table 1 and Table S20). To account for potential variations in defect density, we correlated TOF values with defect concentrations across the three catalysts (Fig. S38). Notably, the observed TOF trend deviates significantly from the estimated defect densities, demonstrating the inherent activity difference between 1D metal–oxo chains and discrete Zr_6_O_8_ nodes. The catalytic performances of commercial Pd/C (Entry 4, Table 1) and Pd@ZrO_2_ (Entry 5, Table 1) are also tested, exhibiting high conversion rates but low selectivity of only 30.6% and 34.4% toward *p*-creosol, respectively, consistent with performances from previously reported work. For the sake of parallel performance comparison, corresponding TOF values based on high conversion rates are further calculated for both Pd@Zr–BTB and Pd@Hf–BTB, which surpass most reported results summarized in Table S19.

**Table 1.** Performance summary of hydrodeoxygenation of vanillin and related compounds 
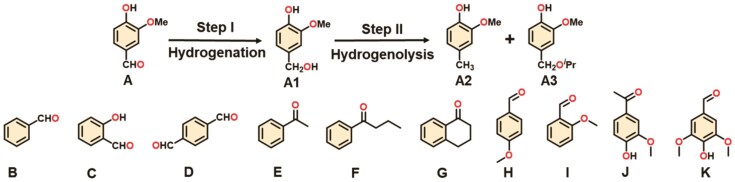

							Selectivity (%)				
Entry	Catalyst	Substrate	T (°C)	H_2_ pressure (MPa)	Time (h)	Conversion (%)	X1	X2	X3	TOF^a^ (h^−1^)	TOF^b^ (h^−1^)
1	Pd@UMCM-309	A	60	0.5	0.75	44.2	78.5	5.9	15.6	16.8	282.5
2	Pd@Zr–BTB	A	60	0.5	0.75	>99.9	0.0	99.0	1.0	639.9	1199.1
3	Pd@Hf–BTB	A	60	0.5	0.75	>99.9	0.1	97.9	2.0	632.8	1044.9
4	Pd@C	A	60	0.5	0.75	91.2	58.0	30.6	11.4	147.9	1153.0
5	Pd@ZrO_2_	A	60	0.5	0.75	94.5	55.2	34.4	10.3	210.0	1124.1
6	Pd@Zr–BTB	B	60	0.5	0.75	>99.9	0.0	99.8	0.2	645.0	1147.2
7	Pd@Zr–BTB	C	60	0.5	0.75	>99.9	0.0	99.1	0.9	640.5	1008.9
8	Pd@Zr–BTB	D	60	0.5	1.5	>99.9	0.0	98.6	1.4	318.6	720.6
9	Pd@Zr–BTB	E	60	0.5	1.0	>99.9	0.0	99.6	0.4	482.8	631.3
10	Pd@Zr–BTB	F	60	0.5	1.0	>99.9	0.0	99.5	0.5	482.3	711.0
11	Pd@Zr–BTB	G	60	0.5	1.0	>99.9	0.0	99.7	0.3	483.3	695.4
12	Pd@Zr–BTB	H	60	0.5	1.0	>99.9	0.0	99.7	0.3	483.3	706.2
13	Pd@Zr–BTB	I	60	0.5	1.0	>99.9	0.0	98.7	1.3	478.5	576.5
14	Pd@Zr–BTB	J	60	0.5	2.0	>99.9	0.3	96.6	3.1	234.1	420.8
15	Pd@Zr–BTB	K	60	0.5	4.0	99.8	2.9	95.4	1.7	115.4	634.1

a TOF was calculated based on corresponding conversion in this table. ^b^The kinetic TOF was calculated based on low conversion (∼20%) in Table S20.

In order to demonstrate the generality of performance of these infinite metal–oxo MOFs, catalytic hydrodeoxygenations for 10 vanillin-like substrates were further explored under the same temperature and hydrogen pressure. As results in Table 1 (Entries 6–15) show, the representative Pd@Zr–BTB catalyst consistently achieves a nearly complete conversion rate (∼99.0%) for all tested substrates. More significantly, the catalytic selectivity toward the corresponding high-value-added hydrodeoxygenated products is also maintained above 95.0%. In short, the reported Pd@Zr–BTB featuring a metal–oxo chain secondary structure has shown both high catalytic activity and selectivity toward the hydrodeoxygenated products. Moreover, the catalytically recyclable experiment was also implemented with both Pd@Zr–BTB and Pd@UMCM-309 catalysts to assess their performance stability. Over 10 consecutive reaction cycles, Pd@UMCM-309 always presents an inferior conversion rate and poor *p*-creosol selectivity. In sharp contrast, Pd@Zr–BTB still holds a substrate conversion rate of >99.0% and >96.0% selectivity toward target *p*-creosol, even after 10 reaction cycles, thanks to its robust 1D metal–oxo structures (Fig. S31). Complementary characterization of recycled Pd@Zr–BTB, including PXRD (Fig. S33), SEM (Fig. S35), BET surface area (Fig. S32) and Pd leaching analysis (Table S21), collectively demonstrates retention of crystallinity, morphology, porosity and compositional integrity. This underscores the Zr–oxo chains’ critical function in stabilizing the framework and shielding Pd NPs. In contrast, after the recycling test, Pd@UMCM-309 shows significant aggregations and crystallinity deterioration (Figs S34 and S36).

**Figure 5.**
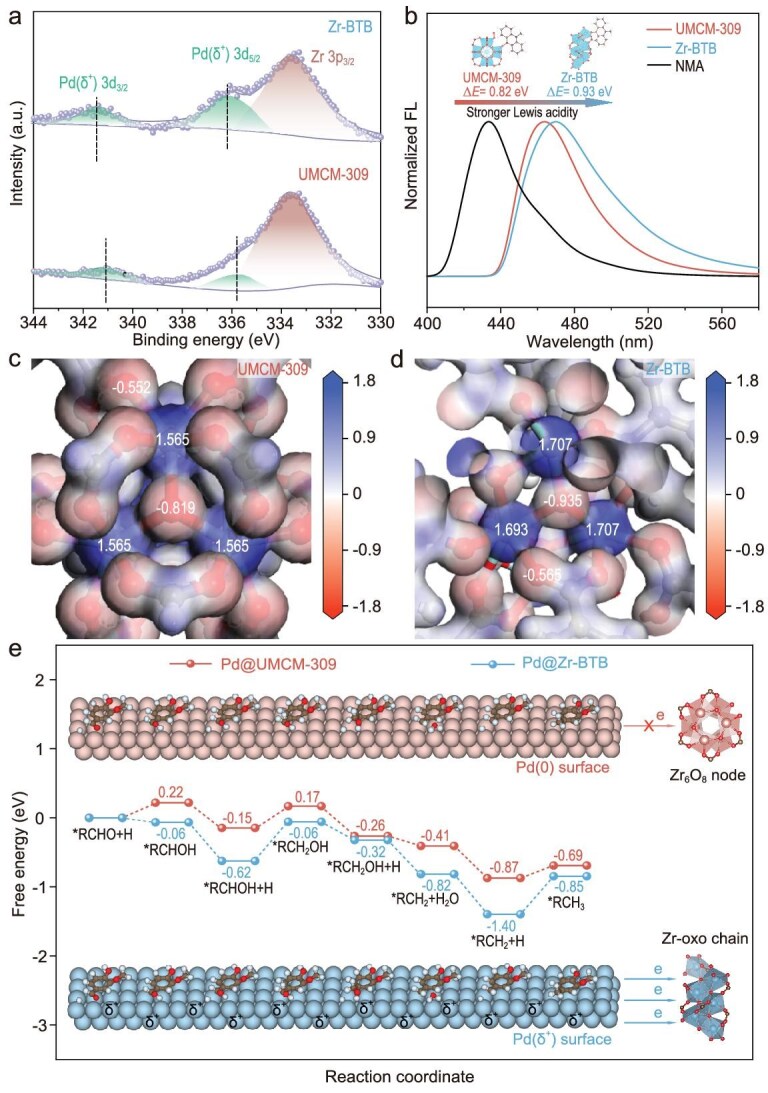
Catalytic mechanism study of Pd@Zr–BTB and Pd@UMCM-309. (a) XPS spectra of Pd@Zr–BTB and Pd@UMCM-309. (b) Fluorescence (FL) spectra of NMA probe upon binding to Zr–BTB and UMCM-309. (c) Charge density distribution profiles of UMCM-309. (d) Charge density distribution profiles of Zr–BTB. (e) Free energy profiles of intermediates of vanillin hydrogenation to vanillyl alcohol and *p*-creosol.

To gain an in-depth mechanistic understanding of the superior catalytic performances achieved by Pd@Zr–BTB, we first employed X-ray photoelectron spectroscopy (XPS) to probe electronic states of tested catalysts, which are considered to be closely charged with the catalytic activation process [49,50]. As shown in Fig. 5a, coupled cyan peaks centered at the binding energy (BE) around 341.0 and 336.0 eV are assigned to the characteristic 3d_3/2_ and 3d_5/2_ of metallic Pd^0^ species, respectively, further confirming successful reductive generation of Pd NPs within MOF samples. Due to spectral overlap between Zr 3p_3/2_ and Pd 3d_5/2_, we hence focus on correlated Pd 3d_3/2_ peaks for clear inspection. Obviously, Pd@Zr–BTB holds a higher BE number (341.5 eV) regarding its Pd 3d_3/2_ peak in comparison with Pd^0^ (341.0 eV) of Pd@UMCM-309, demonstrating a remarkable electron transfer from Pd NPs to 1D metal–oxo chains of Zr–BTB [51]. Crucially, such electron-deficient Pd states via metal–support interactions (MSIs) correlated with the superior catalytic performances in hydrodeoxygenation of vanillin according to a well-studied reaction mechanism [52,53]. To assess electron regulation disparity, we compare the electrophilicity (i.e. Lewis acidity) of correlated metal nodes using a prototypical *N*-methylacridone (NMA)-based fluorescent assay [54]. As the control, free NMA emits at 433.0 nm (Fig. 5b), which will shift toward a higher wavelength upon binding to electrophilic metal nodes. In principle, the metal node type of a higher Lewis acidity in MOFs strengthens NMA binding and therefore induces a larger redshift of the emission peak. The observed emission peak centered at 470.0 nm for Zr–BTB is clearly more redshifted than the 463.0 nm observed for UMCM-309, demonstrating the stronger Lewis acidity of Zr–BTB over UMCM-309. Analogous behavior is observed in comparative analysis of Hf–BTB and UMCM-309(Hf), further confirming enhanced Lewis acidity at the metal–oxo sites (Fig. S37).

We then calculated the charge location of different metal nodes in Zr–BTB and UMCM-309 MOFs and depict corresponding electronic density profiles in Fig. 5c and d for understanding the electrophilicity disparity at the molecular level. Each Zr site of Zr_6_O_4_(μ_3_-OH)_4_ node in conventional UMCM-309 presents a Mulliken charge of +1.565, which is balanced by negatively charged carboxylate oxygens (−0.552) and oxo-bridged oxygens (μ_3_-OH, −0.819). Remarkably, the Zr site of the 1D [Zr(μ_3_-O)]_∞_ chain of Zr–BTB occupies a much higher Mulliken charge of +1.707, balanced by similar carboxylate oxygens (−0.565) and distinct oxo-bridged μ_3_-O (−0.935). This shows that the stronger electronegativity of distinct μ_3_-O compared to μ_3_-OH accounts for the higher Lewis acidity of metal–oxo Zr–BTB over conventional UMCM-309.

Based on distinct electronic states of Pd NPs in Pd@UMCM-309 versus Pd@Zr–BTB, we further examine their essential roles in modulating energy profiles of key intermediates during catalytic pathways using density functional theory (DFT) calculations. In good agreement with previously reported results [52,55], our computed catalysis pathway initiates with vanillin adsorption onto Pd surfaces and experiences four main intermediates to form hydrodeoxygenated *p*-creosol (Fig. 5e). Specifically, (i) the reaction between dissociated hydrogen and adsorbed vanillin (denoted *RCHO) forming an *RCHOH intermediate; (ii) the addition of a second hydrogen yielding a hydrogenated *RCH_2_OH product; (iii) the deoxygenation of *RCH_2_OH to an *RCH_2_ intermediate; and (iv) the final hydrogen addition forming the hydrodeoxygenated *RCH_3_ product. To highlight MOFs’ electron-transfer effect, Pd surfaces with distinct electronic states are modeled separately for UMCM-309 and Zr–BTB. Along with the reaction coordinate on Pd surfaces with negligible electron transfer (Pd@UMCM-309, red pathway in Fig. 5e), it is thermodynamically unfavorable for proceeding hydrogenation of adsorbed *RCHO into *RCHOH, as well as subsequent *RCH_2_OH intermediates, as supported by the endothermic energies of 0.22 and 0.17 eV, respectively. The computational result is also consistent with the low catalytic activity of Pd@UMCM-309. Conversely, the reaction coordinate on Pd surfaces with populations of partial positive charge via electron transfer to the Zr–oxo node (Pd@Zr–BTB, blue pathway in Fig. 5e) presents a barrier-free catalytic pathway (an exothermic energy of −0.06 eV for both *RCHO into *RCHOH intermediates), theoretically explaining the superior catalytic activity of Pd@Zr–BTB. Moreover, the thermodynamic energy for formation of target *RCH_3_ product in the case of Pd@Zr–BTB is calculated to be −0.85 eV, which is lower than −0.69 eV in the case of Pd@UMCM-309. Hence the much more favorable thermodynamic pathway in producing target *RCH_3_ product also supports the excellent *p*-creosol selectivity achieved by Pd@Zr–BTB over Pd@UMCM-309.

## CONCLUSION

In this work, we have established an unconventional acetic acid-based solvothermal protocol for engineering a novel 1D [Zr(μ_3_-O)]_∞_ chain-based Zr–BTB MOF with an unambiguously identified structure by SXRD characterization. The protocol demonstrates broad generality, successfully extending to isoreticular Hf–BTB and Ce–BTB analogs, as well as the Zr(μ_3_-O) (4F-BDC) and [Zr(μ_3_-O)]_2_(TCPP) MOFs utilizing diverse carboxylate linkers. In catalytic hydrodeoxygenation upgrading of natural feedstocks to high-caloric liquid fuels, Zr–BTB-derived catalyst exhibits both high catalytic activity (99.9%) and product selectivity (>95.0%) towards 10 kinds of substrate, attributed to abundant open Zr(IV) sites with strong Lewis acidity. Strikingly, the conventional Zr_6_O_8_ node-based UMCM-309 counterpart shows poor conversion and even fails to generate target hydrodeoxygenated products. Furthermore, the robust [Zr(μ_3_-O)]_∞_ chain endows Zr–BTB with exceptional performance durability, maintaining undiminished catalytic activity and product selectivity after 10 successive cycles. In light of our results, it is foreseeable that phase engineering of higher-dimensional metal–oxo units, including 1D chains and even 2D sheets in MOFs, can unlock unprecedented properties and functionalities for versatile applications beyond catalysis.

## Supplementary Material

nwag018_Supplemental_Files
