# Supplementary material for: A general protocol for engineering metal–oxo-chain standing frameworks
Source: Natl Sci Rev. 2026 Jan 15;13(5):nwag018. doi: 10.1093/nsr/nwag018 (PMC12980333; doi:10.1093/nsr/nwag018)
Supplement: nwag018_Supplemental_Files [file nwag018_supplemental_files.zip › 1958-Supporting Information.pdf]

A General Protocol for Engineering Metal-oxo-chain Standing Frameworks

Jun Guo,*[a] Zhiyong Ban,[a] Yutian Qin,[b] Siyang Li,[c] Zelong Zhao,[a] Yongli Ji,[a] Peter E. VanNatta,[d] Yin Zhang,*[d] Meiting Zhao,*[b] Thamraa AlShahrani,[e] and Shengqian Ma*[d]

[a]State Key Laboratory of Separation Membranes and Membrane Processes, School of Electronics and Information Engineering & School of Chemistry, Tiangong University, Tianjin 300387, P. R. China.

[b]Tianjin Key Laboratory of Molecular Optoelectronic Sciences, Department of Chemistry, Institute of Molecular Aggregation Science, Tianjin University, Tianjin 300072, P. R. China.

[c]CAS Key Laboratory of Nanosystem and Hierarchical Fabrication, CAS Center for Excellence in Nanoscience, National Center for Nanoscience and Technology, Beijing 100190, P. R. China.

[d]Department of Chemistry, University of North Texas, Texas 76201, USA.

[e]Department of Physics, College of Science, Princess Nourah bint Abdulrahman University, Riyadh 11564, Saudi Arabia.

*Corresponding authors. shengqian.ma@unt.edu; junguo@tiangong.edu.cn; yin.zhang@unt.edu; mtzhao@tju.edu.cn

MATERIALS

Zirconium(IV) chloride ($ZrCl_4$, >99.9%), hafnium(IV) chloride ($HfCl_4$, 99.5%), ammonium ceric (IV) nitrate [$(NH_4)_2Ce(NO_3)_6$, 99%], 1,3,5-Tris(4-carboxyphenyl)benzene (BTB, >97.0%), 2,3,5,6-tetrafluoroterephthalic acid (4F-BDC, 97.0%), 4,4',4''-(2,4,6-trimethylbenzene-1,3,5-triyl)tribenzoic acid (TCPP, >97.0%), *N*,*N*-dimethylformamide (DMF, >99.9%), 96.0 wt.% sulfuric acid-d_2 in D_2O (D_2SO_4, D. 99.5%), dimethyl sulfoxide-d_6 (DMSO, D. 99.9%) were bought from Innochem Beijing Co. Ltd. 4-Hydroxy-3-methoxybenzaldehyde (98.0%), benzaldehyde (98.0%), salicylaldehyde (98.0%), terephthalaldehyde (98.0%), acetophenone (98.0%), butyrophenone (98.0%), α-tetralone (98.0%), 4-methoxybenzaldehyde (98.0%), 2-methoxybenzaldehyde (98.0%), methoxyacetophenone (98.0%), 3,5-dimethoxy-4-hydroxybenzaldehyde (98.0%), glycidyl phenyl ether (>99.0%) and *N*-methylacridone (98%) were purchased from Aladdin Reagent Co. Ltd. Acetic acid (>99.0%), H_2PdCl_4 (>99.0%) and concentrated chloride acid (HCl, 12.0 mol/L) were bought from Sigma-Aldrich. Methanol (99.9%), ethanol (99.9%), isopropanol (99.9%), acetonitrile (99.9%) and acetone (99.9%) were purchased from RCI Labscan. All chemicals were used as received without further purification. The deionized water (18.2 $M\Omega \cdot cm^{-1}$ resistivity) was obtained from the Milli-Q System.

CHARACTERIZATION

Scanning electron microscopy (SEM) characterization was performed with Quattro S (Thermo Scientific). High-resolution transmission electron microscopy (HR-TEM) was observed with JEO JEM-2100F microscopy. The HAADF-STEM images were observed with spherical aberration electron microscopy (JEOL JEM-ARM300F). Fourier-transform infrared (FT-IR) data were collected using a Nicolet Avatar 360 FT-IR spectrometer. X-ray photoelectron spectroscopy (XPS) characterization was performed with NEXSA (Thermo Fisher). Fluorescence (FL) data were collected by using Gangdong F-320 fluorescence spectrophotometer. Nuclear magnetic resonance (NMR) spectra were obtained on a 600 MHz Bruker superconducting-magnet high-field equipment. Thermogravimetric analysis (TGA) was conducted on PerkinElmer equipment (STA 6000) under nitrogen with a heating rate of 10°C/min. The palladium loading among different samples were analyzed by inductively coupled plasma optical emission spectrometry (ICP-OES) using an Agilent 5110 instrument. Pore size distributions were calculated by applying the non-local density functional theory (NLDFT) equilibrium model assuming a slit-shaped pore. Powder x-ray diffraction (PXRD) plots were recorded on a Bruker D2 Phaser diffractometer (Cu Kα radiation, λ = 1.5406 Å) equipped with a LYNXEYE XE-T detector. Single-crystal characterization of Zr-BTB was conducted on a Bruker APEX-II CCD detector with Ga Kα X-ray source (λ = 1.34139 Å). Single crystal characterization of Hf-BTB was collected by a diffractometer Rigaku Oxford Diffraction Supernova Dual Source, Cu at Zero equipped with an AtlasS2 CCD using Cu Kα radiation (λ = 1.54178 Å) by using a w scan mode. The structure was solved with the SIR2004 structure solution program using Direct Methods and refined with the XL refinement package using Least Squares minimization.

METHODS

The growth of Zr-BTB MOF single crystals.

The growth of Zr-BTB single crystal was conducted via an acetic acid-based solvothermal protocol. Typically, 117.0 mg (0.5 mmol) of $ZrCl_4$ and 87.6 mg (0.2 mmol) of BTB were added to 10 mL of acetic acid. The resulting mixture was sonicated for 5 minutes and then transferred to a 25 mL stainless autoclave. The autoclave was sealed tightly and placed into a 220°C oven for heating over 96 hours. After cooling to room temperature, the autoclave was opened, and crystals were collected carefully for further characterization.

The growth of Hf-BTB MOF single crystals.

The growth of Hf-BTB single crystal was conducted similarly except for using 160.0 mg (0.5 mmol) of $HfCl_4$ as the precursor.

The activation procedure of MOFs.

MOF catalysts were activated according to the procedure reported in the literature.[1] First, each MOF catalyst was immersed in 50 mL of methanol at 60°C for one day during which fresh methanol was replaced twice. The precipitates were collected by centrifuging at 10000 r.p.m for 5 minutes and then washed with 30 mL of acetone 3 times. Finally, the resulting MOF catalyst was dried at 60°C overnight for further use and test.

Preparation of Pd@MOF catalysts.

Pd@Zr-BTB, Pd@Hf-BTB and Pd@UMCM-309 were prepared via a reported double-solvent method.[2] In a 25 mL round-bottomed flask, 30 mg of MOFs were dispersed in n-hexane (10 mL) with vigorous stirring. An aqueous solution of H_2PdCl_4 (0.1 mol/L, 29 μL) was then added to the dispersion. After stirring for 12 h, n-hexane was removed, and the residual slurry was dried in a vacuum oven at 50°C. The resultant powder was reduced in H_2/Ar (10%, v/v) atmosphere at 200°C for 4 hours with a ramp rate of 5°C/min.

Catalytic hydrodeoxygenation of natural feedstocks.

A mixture containing substrate (0.15 mmol), isopropanol (4 mL), and Pd@MOF catalyst (4 mg) was placed in a 20-mL PTFE-lined stainless-steel reactor. The reactor was charged with H_2 (0.5 MPa), sealed and kept at 60°C with 500 r.p.m. magnetic stirring. After the reaction, the catalyst was collected via centrifugation (8000 rpm) for 5 minutes and washed with fresh isopropanol for subsequent catalytic runs. The conversion and selectivity were analyzed with gas chromatography (Shimadzu 2010 Plus, equipped with 0.25 mm × 30 m Rtx-5 capillary column).

NMA-based fluorescence probing.

According to the established protocol,[3] the FL spectrum of pristine NMA solution dissolved in MeCN was measured under an excitation light of 413 nm. In order to probe the Lewis acidity of MOF, 5 mg of MOF sample was added to 4 mL of 10 μM NMA solution in MeCN. The resulting mixture was sonicated for 2 minutes and then transferred to a fluorescence cuvette for FL measurement. The excitation light was also set to 413 nm.

Pore size theoretical estimations.

The pore size distribution of ideal Zr-BTB and Hf-BTB crystals was calculated based on the Voronoi decomposition algorithm packaged in the Zeo++ suite free of charge.[4] In detail, the typical Voronoi decomposition protocol is adapted to describe the pore geometry. The hydrogen molecule with a radius of 0.11 nm is selected as the probe and 100,000 sample points are taken in the near-surface of frameworks.

The calculation of charge distribution of MOF.

The charge distribution calculation was performed through the Dmol3 Module integrated into the Material Studio software package.[5] The corresponding MOF single-crystal structure was used as a model. The electric charge density of the metal node was calculated by the generalized gradient approximation (GGA)-Perdew-Burke-Ernzerhof (PBE) functional method with convergence tolerance set at fine precision.[6]

Catalytic reaction coordinate based on DFT simulations.

Density functional theory (DFT) calculations were performed using the Vienna Ab initio Simulation Package (VASP).[7-10] Exchange and correlation energy is described by the Perdew-Burke-Ernzerhof (PBE) functional[7,8] within the generalized gradient approximation (GGA). The electron-ion interactions are calculated using the projected augmented wave (PAW) method[11]. A first-order Methfessel-Paxton method with a smearing of 0.1 eV is chosen to determine the partial occupancies of Kohn-Sham orbitals. The cutoff energy of the plane waves basis set was 400 eV throughout all calculations except the bulk optimization, where a higher cutoff of 600 eV is used.

We simplify our catalysts into slab models of Pd species in DFT calculations. For Pd@UMCM-309, a 4-layered $(6\times3\sqrt{3})$ Pd (111) supercell is constructed. To simulate the positive charge of Pd species in Pd@Zr-BTB, a 3-layered (2×5) PdO (101) supercell is built. All periodic slabs have a vacuum layer of at least 15 Å. A 2×2×1 Gamma centered *k*-point mesh is employed in the structural relaxation. For all slab models, the bottom layer is fixed to represent the bulk phase while the rest parts are fully relaxed. The convergence tolerances for electronic and ionic steps are set to be 10^{-5} eV and 0.05 eV $Å^{-1}$. The long-range dispersion interaction is described by the DFT-D3 method.[12] The solvation effect of isopropanol is considered for the refinement of energies by using the VASPsol module.[13,14] To obtain the correction items for calculating Gibbs free energy, vibration analysis is performed. The results are analysed using the VASPKIT program.[15] The adsorption energy of reaction intermediates is computed using the following Equation (1):

$$\Delta G_{\mathrm{ads}} = (E_{*\mathrm{ads}} - E_{*} - E_{\mathrm{ads}}) + \Delta E_{\mathrm{ZPE}} - T\Delta S \#(1)$$

Where ads = RCHO + H, RCHOH, RCHOH + H, RCH_2OH, RCH_2OH+H, RCH_2 + H_2O, RCH_2 + H, and RCH_3 (R = $C_7H_7O_2$ and RCHO represents the vanillin molecule). In the equation above, ΔE_{ZPE} is the zero-point energy change, ΔS is the entropy change. In this work, the values of ΔE_{ZPE} and ΔS are obtained from the vibration frequency analysis setting $T = 333.15$ K (i.e. the catalysis temperature implemented in this work).

The synthesis of Zr-BTB MOF powders.

Typically, 234.0 mg (1.0 mmol) of $ZrCl_4$ and 175.2 mg (0.4 mmol) of BTB were added to 10 mL of acetic acid. The resulting mixture was sonicated for 5 minutes and then transferred to a 25 mL stainless autoclave. The autoclave was sealed tightly and placed into a 220°C oven for heating over 36 hours. After cooling to room temperature, the autoclave was opened, and the precipitates were collected by centrifuging at 8000 r.p.m for 5 minutes. After washing with 30 mL of DMF 3 times, the obtained products were further exchanged with 30 mL of acetone 3 times in order to exchange DMF with volatile acetone. Finally, the products were evacuated at 60°C overnight for further use. The yield of weighted Zr-BTB powder is 81%.

The synthesis of Hf-BTB MOF powders.

The synthesis of Hf-BTB powder was conducted similarly except for using 320.0 mg (1.0 mmol) of $HfCl_4$ as the precursor. The yield of weighted Hf-BTB powder is 77%.

The synthesis of Ce-BTB MOF.

BTB (123.1 mg, 0.28 mmol) and $(NH_4)_2Ce(NO_3)_6$ (97.1 mg, 0.18 mmol) were added to 4.0 mL of acetic acid. The mixture was treated under sonication for 20 minutes and then transferred into a 25 mL stainless autoclave. Then, the autoclave was sealed tightly and heated at 140°C for 10 hours. After cooling to room temperature, the autoclave was opened, and products were collected for further characterization. The yield of weighted Ce-BTB powder is 66%.

The synthesis of 1D Zr-oxo chain MOF by ditopic linker (MIL-140-4F).

2,3,5,6-Tetrafluoroterephthalic acid (238 mg, 0.1 mmol) and $ZrCl_4$ (120.0 mg, 0.5 mmol) were added to 10.0 mL of acetic acid. The mixture was treated under sonication for 20 minutes and then transferred into a 25 mL stainless autoclave. Then, the autoclave was sealed tightly and heated at 220°C for 48 hours. After cooling to room temperature, the autoclave was opened, and products were collected for further characterization. The yield of weighted MIL-140-4F powder is 72%.

The synthesis of 1D Zr-oxo chain MOF by tetratopical linker (PCN-226).

Typically, 92.0 mg (0.4 mmol) of $ZrCl_4$ and 79.0 mg (0.1 mmol) of TCPP were added to 10 mL of acetic acid. The resulting mixture was sonicated for 5 minutes and then transferred to a 25 mL stainless autoclave. Then, the autoclave was sealed tightly and placed into a 220^oC oven for 72 hours. After cooling to room temperature, the autoclave was opened, and products were collected for further characterization. The yield of weighted PCN-226 powder is 82%.

The synthesis of UMCM-309 with discrete $Zr_6(\mu_3\text{-}O)_4(\mu_3\text{-}OH)_4$ node.

UMCM-309 was synthesized according to the reported method[19] with minor modifications. Firstly, 70.2 mg (0.3 mmol) of $ZrCl_4$ and 109.0 mg (0.25 mmol) of BTB were added into a mixed solvent composed of 5 mL of DMF and 5 mL of concentrated HCl (36-38% wt.). The obtained mixture was sonicated for 5 minutes and then transferred to a 25 mL stainless autoclave. The autoclave was sealed tightly and placed in a 120^oC oven for 48 hours. White precipitates were collected by centrifuging at 10000 r.p.m for 5 minutes and washed with 30 mL of DMF 3 times. The obtained products were then further washed with 30 mL of acetone 3 times in order to exchange DMF with volatile acetone. Finally, the products were evacuated at 60^oC overnight for further use. The yield of weighted UMCM-309 powder is 81%.

SUPPLYMENTARY FIGURES

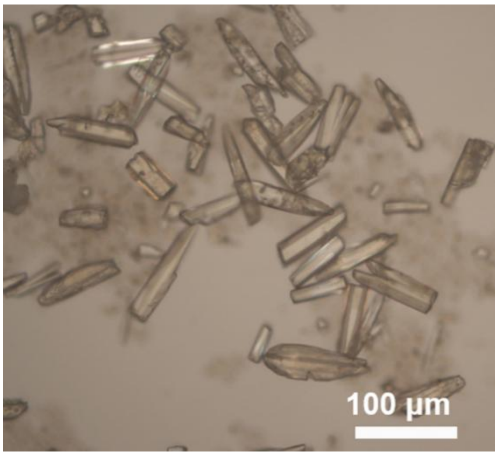

Figure S1. The optical image of Zr-BTB crystals.

The resultant Zr-BTB single crystals cultivated in acetic acid at 220°C show rod-shaped morphologies.

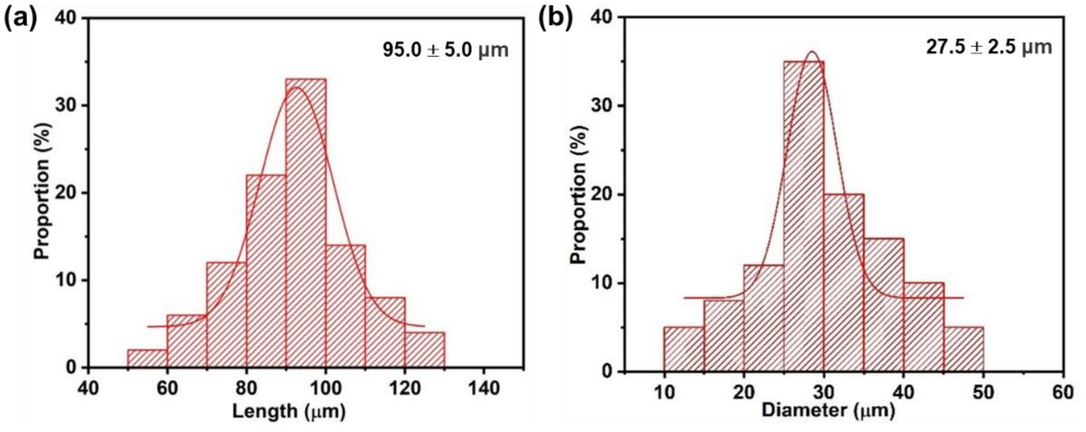

Figure S2. The size counting of Zr-BTB crystals.

The obtained Zr-BTB crystals show an average length of 95.0 μm (Figure S2a) and an average diameter of 27.5 μm (Figure S2b).

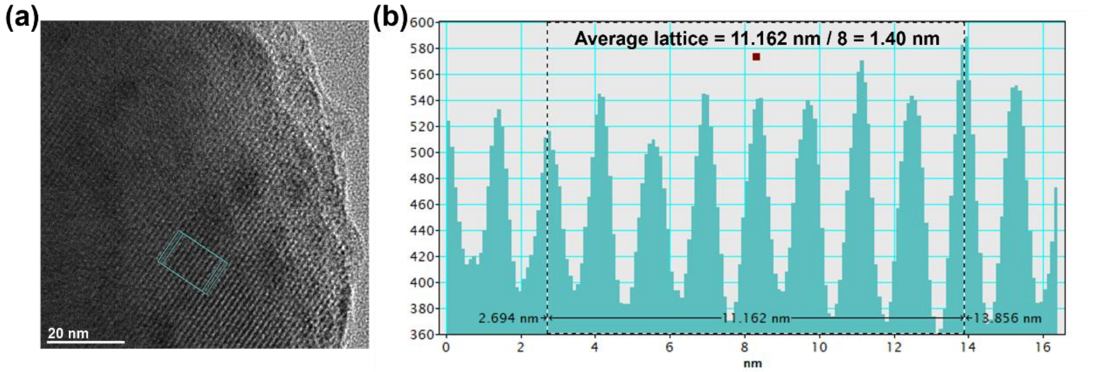

Figure S3. The HR-TEM image of Zr-BTB.

Clear lattice fringes can be seen in Figure S3a, and the corresponding integrated pixel intensity of the green rectangle region indicates an average lattice of 1.40 nm (Figure S3b).

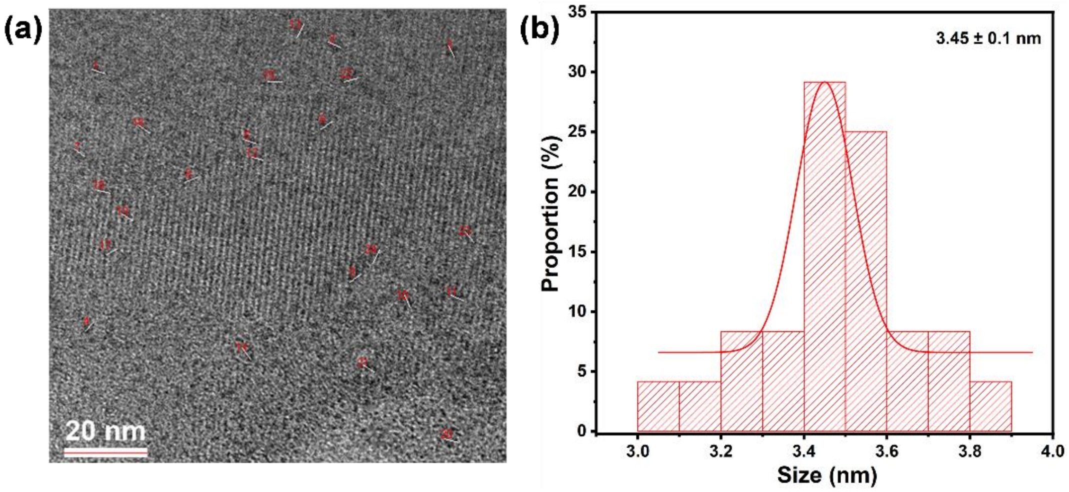

Figure S4. Particle size distribution of Pd NPs of Pd@Zr-BTB.

The average particle size of Pd NPs within Zr-BTB is about 3.5 nm.

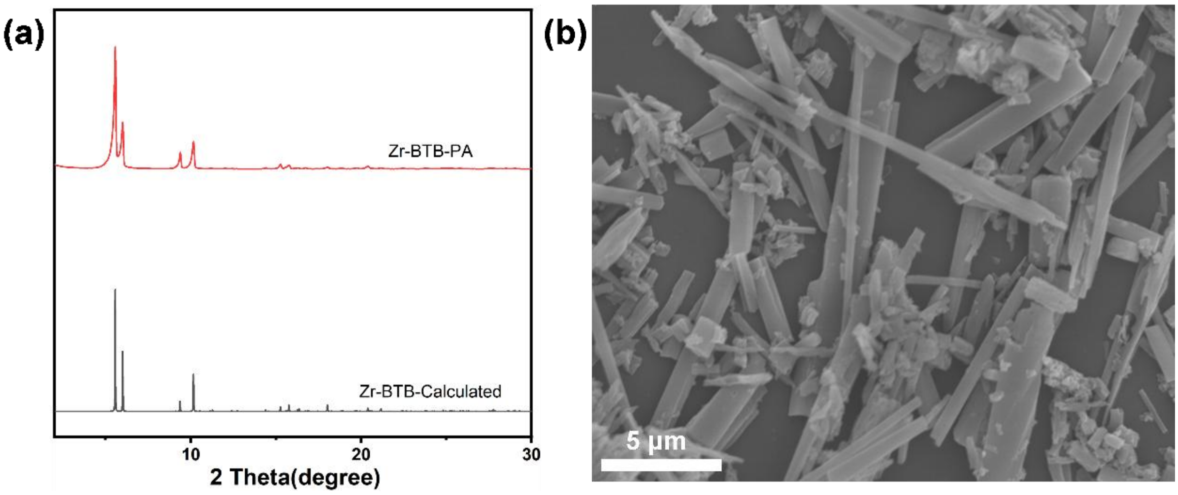

Figure S5. (a) XRD pattern of Zr-BTB-PA; (b) SEM image of Zr-BTB-PA.

The obtained Zr-BTB using propionic acid (PA) as the solvent shows an identical crystallographic structure to calculated result, and Zr-BTB-PA shows rod-shaped morphologies.

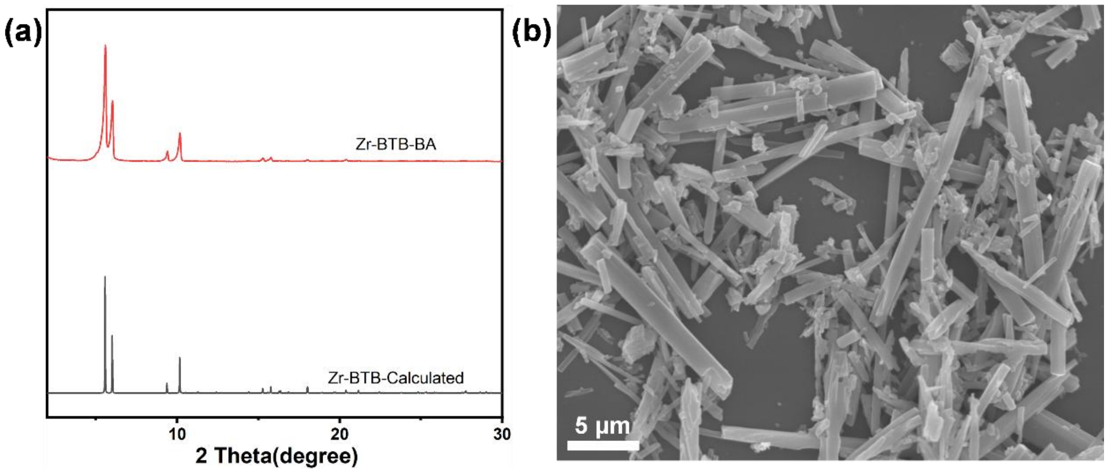

Figure S6. (a) XRD pattern of Zr-BTB-BA; (b) SEM image of Zr-BTB-BA.

The obtained Zr-BTB using butyric acid (BA) as the solvent shows an identical crystallographic structure to calculated result, and Zr-BTB-BA shows rod-shaped morphologies.

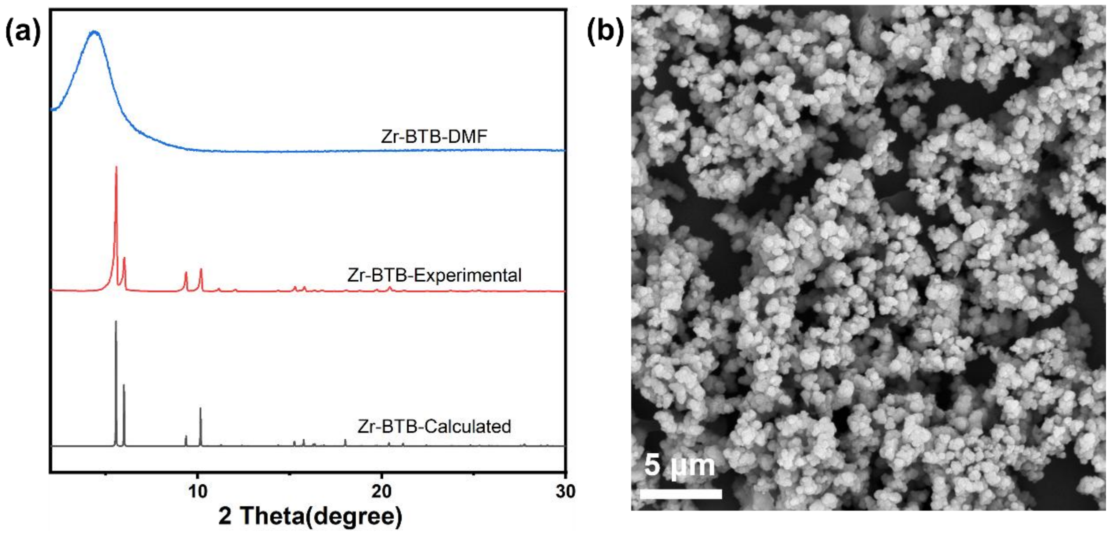

Figure S7. (a) XRD pattern of Zr-BTB-DMF; (b) SEM image of Zr-BTB-DMF.

As shown in Figure S7, PXRD results and SEM image show that the obtained Zr-BTB by using conventional DMF-based solvothermal method is an amorphous product.

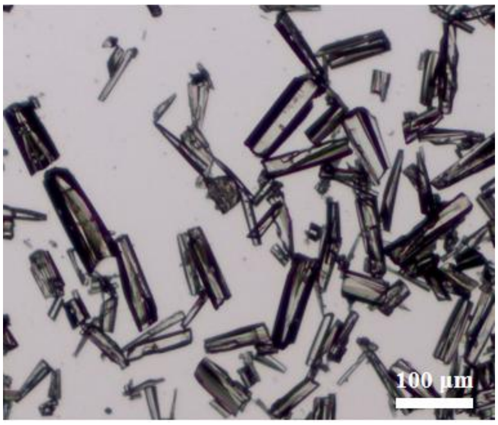

Figure S8. The optical image of Hf-BTB crystals.

The resultant Hf-BTB single crystals cultivated in acetic acid at 220°C also show rod-shaped morphologies.

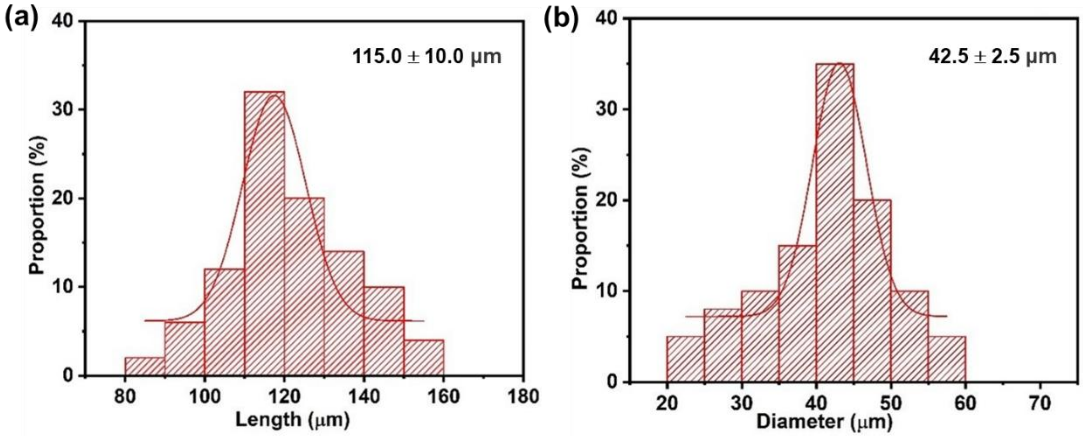

Figure S9. The size counting of Hf-BTB crystals.

The obtained Hf-BTB crystals show an average length of 115.0 μm (Figure S9a) and an average diameter of 42.5 μm (Figure S9b).

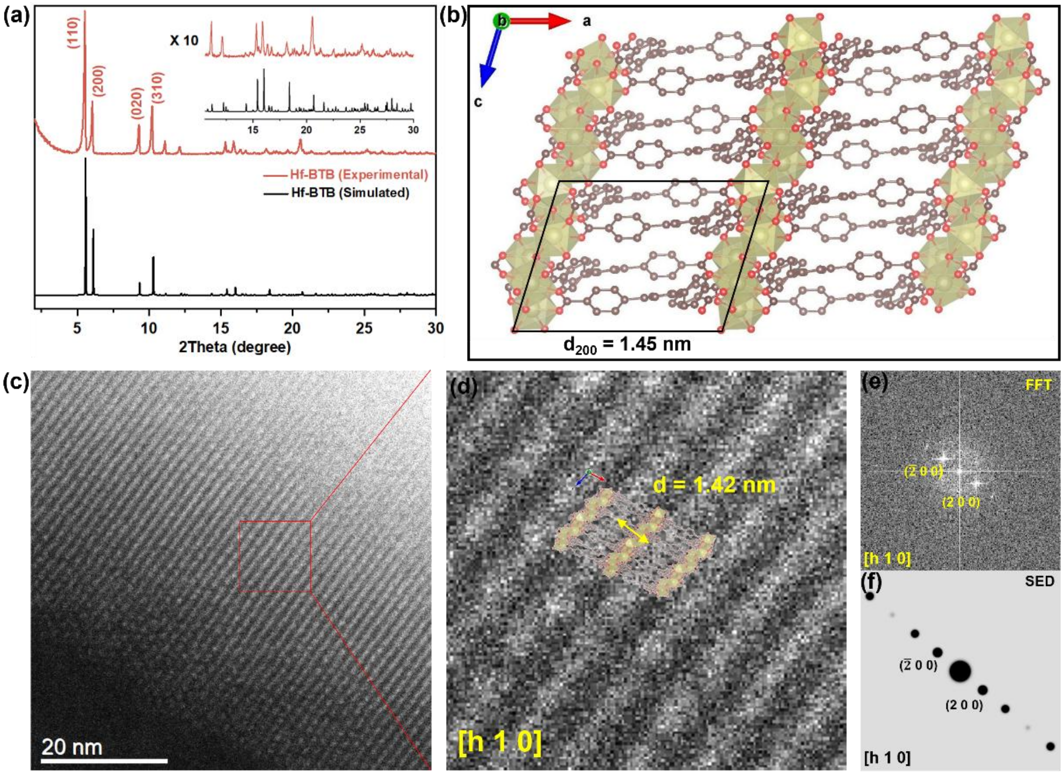

Figure S10. (a) Experimental PXRD pattern of Hf-BTB and corresponding calculated one according to the single-crystal result (CCDC No. 2330779). (b) The crystal structure illustration of Hf-BTB viewed along the *b*-axis. (c) HAADF-STEM characterization of Zr-BTB. (d) Magnification of the red solid rectangle region indicated in (c). (e) Derived FFT pattern of the lattice profile shown in (d). (f) Corresponding SED pattern based on SXRD.

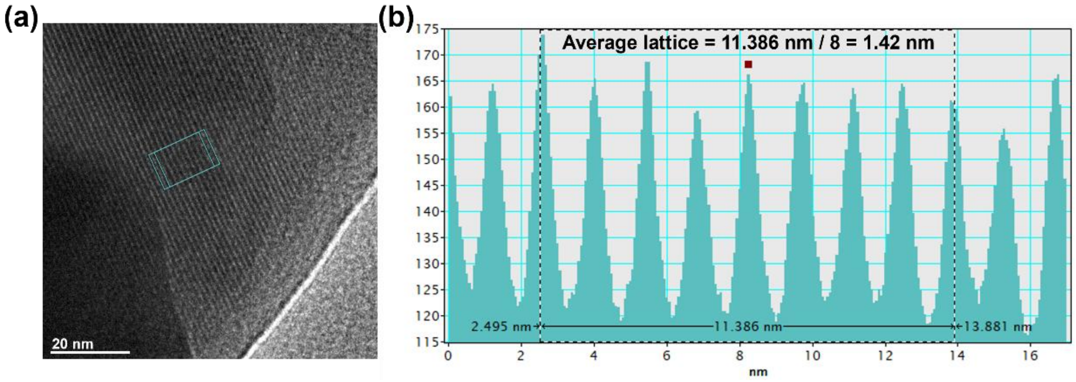

Figure S11. The HR-TEM image of Hf-BTB.

Clear lattice fringes can be seen in Figure S11a for Hf-BTB, and the corresponding integrated pixel intensity of the green rectangle region indicates an average lattice of 1.42 nm (Figure S11b).

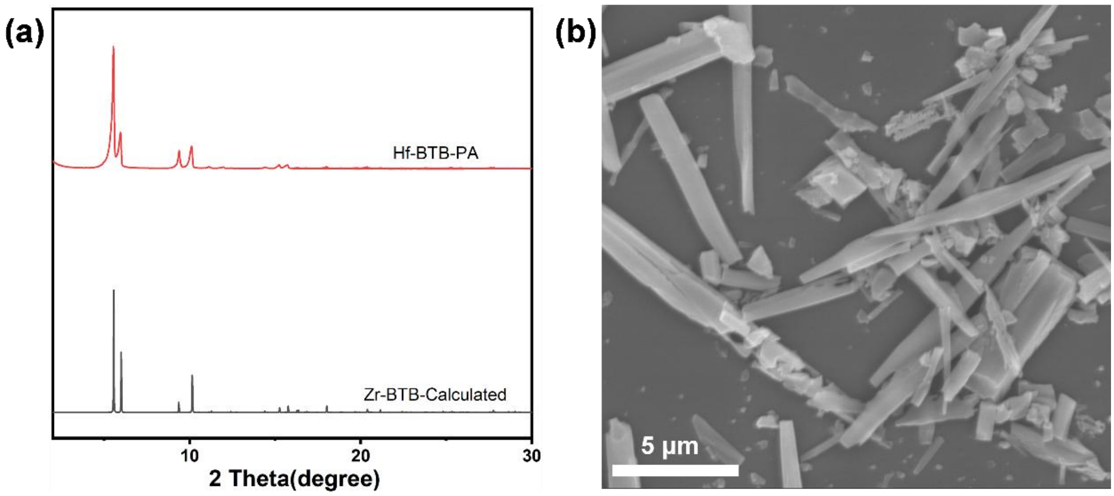

Figure S12. (a) XRD pattern of Hf-BTB-PA; (b) SEM image of Hf-BTB-PA.

The obtained Hf-BTB using propionic acid (PA) as the solvent shows an identical crystallographic structure to calculated result, and Hf-BTB-PA shows rod-shaped morphologies.

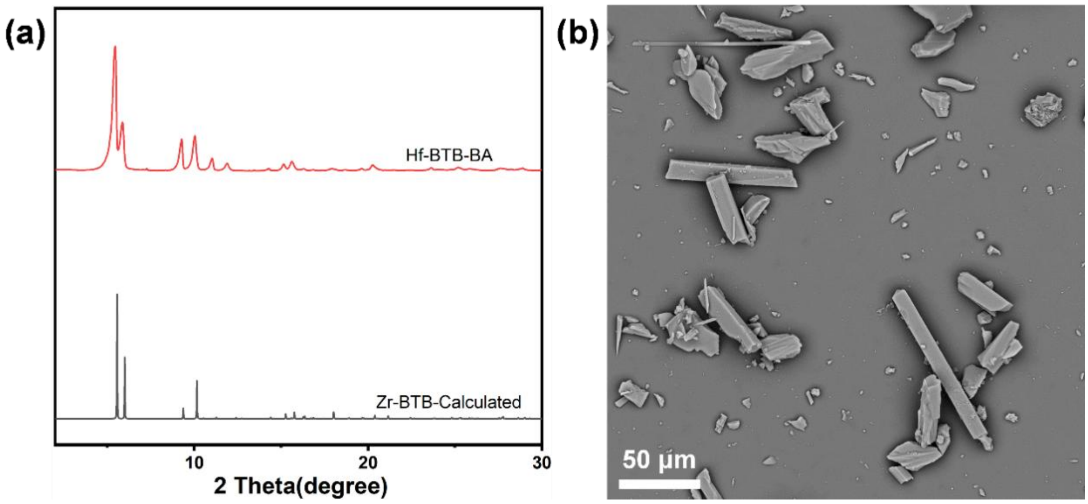

Figure S13. (a) XRD pattern of Hf-BTB-BA; (b) SEM image of Hf-BTB-BA.

The obtained Hf-BTB using butyric acid (BA) as the solvent shows an identical crystallographic structure to calculated result, and Hf-BTB-BA shows rod-shaped morphologies.

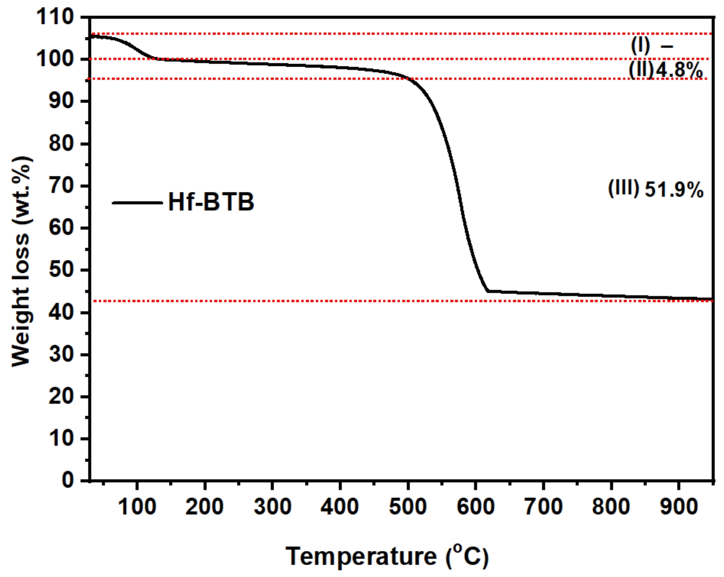

Figure S14. TGA curve of Hf-BTB sample under N_2 atmosphere.

Decomposition process:

(I) Hf_2O_2(BTB)(AC)(Sol)→Hf_2O_2(BTB)(AC)

(II) Hf_2O_2(BTB)(AC)→Hf_2O_2(BTB)(OH)

(III) Hf_2O_2(BTB)(OH)→2HfO_2)

The thermogravimetric analysis of Hf-BTB also shows unusually high thermal stability with a T_d of 542°C.

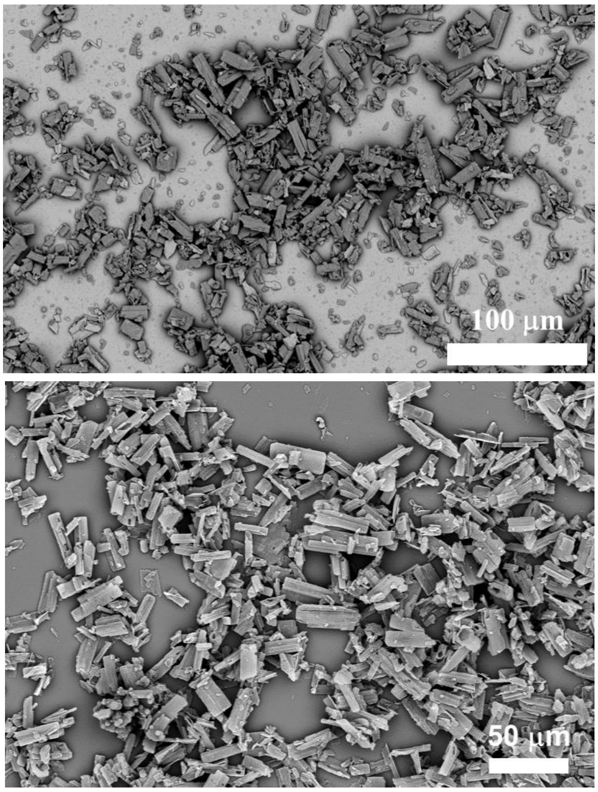

Figure S15. The SEM image of Ce-BTB MOF crystals.

The resultant Ce-BTB single crystals cultivated in acetic acid at 220°C also show rod-shaped morphologies.

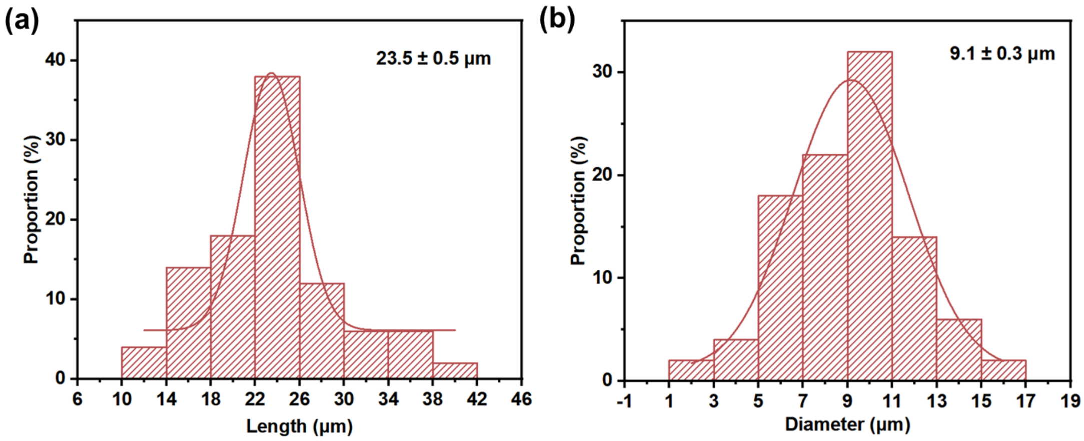

Figure S16. The size counting of Ce-BTB crystals.

The obtained Ce-BTB crystals show an average length of 23.5 μm (Figure S16a) and an average diameter of 9.1 μm (Figure S16b).

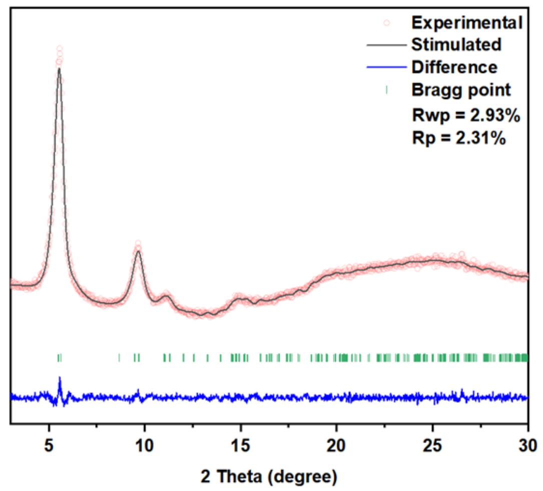

Figure S17. The PXRD refinement patterns of Ce-BTB MOF crystals.

On account of the strong oxidation potential of adopted Ce(IV), the crystal quality of the resultant Ce-BTB is not satisfactory for SXRD identification. We therefore refine the crystallographic parameters (Table S15) based on its isoreticular nature to Zr-BTB/Hf-BTB with well-converged refinement indices.

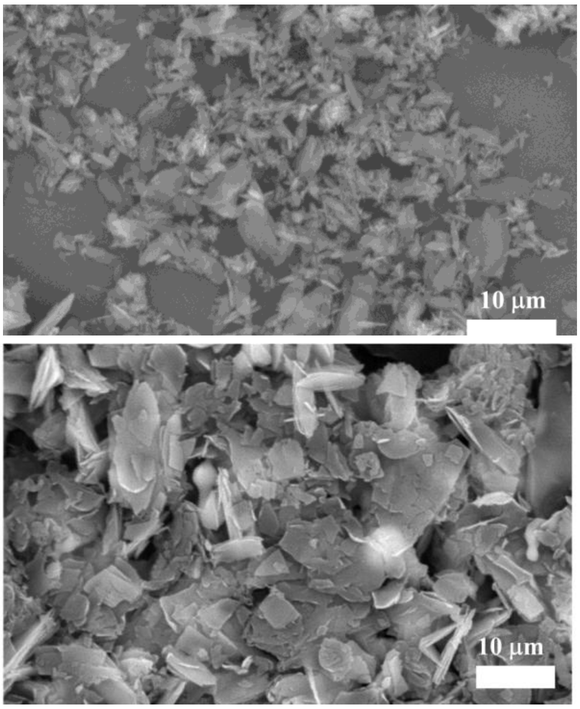

Figure S18. The SEM image of 1D Zr-oxo MOF by changing BTB to 4F-BDC.

The resultant product shows sheet-shaped morphologies using the same acetic acid-based solvothermal protocol.

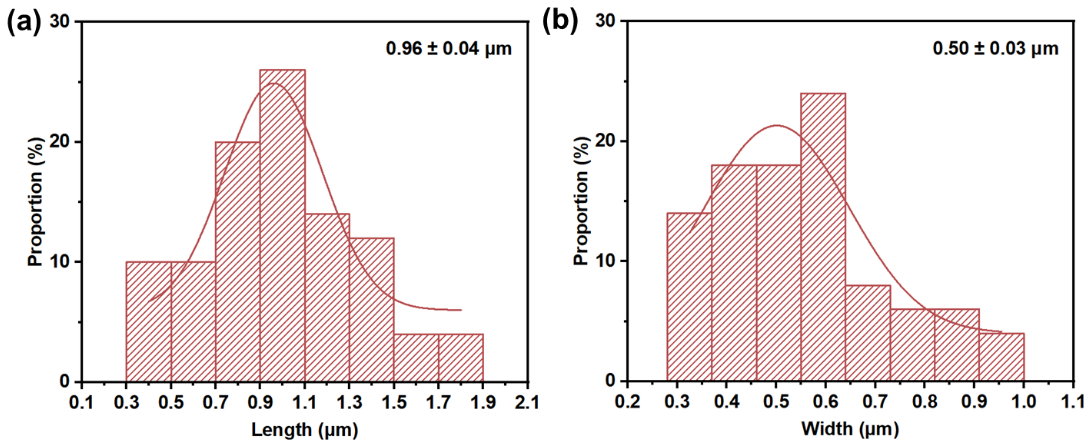

Figure S19. The size counting of 1D Zr-oxo MOF using 4F-BDC as the linker.

The obtained product shows an average length of 0.96 μm (Figure S19a) and an average width of 0.50 μm (Figure S19b).

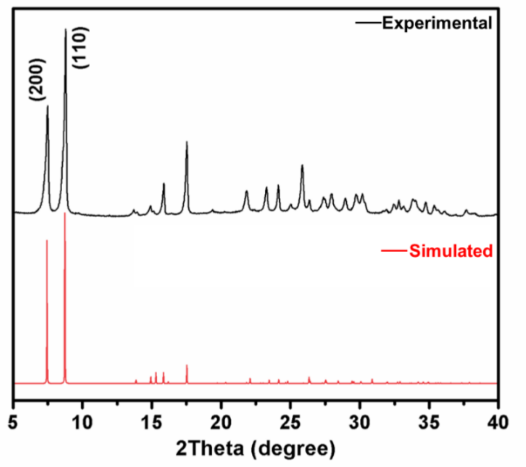

Figure S20. The PXRD of 1D Zr-oxo MOF using 4F-BDC as the linker.

The obtained 1D Zr-oxo MOF using 4F-BDC as the linker shows an identical crystallographic structure to MIL-140-4F reported previously.[17]

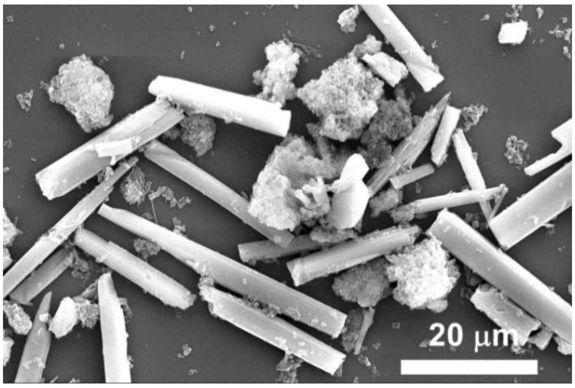

Figure S21. The SEM image of 1D Zr-oxo MOF by changing BTB to tetratopic TCPP.

The resultant product shows rod-shaped morphologies using the same acetic acid-based solvothermal protocol.

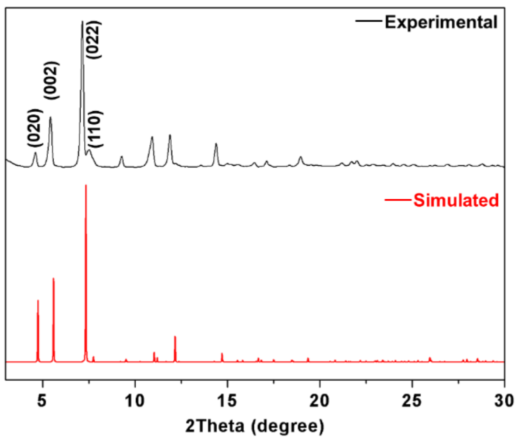

Figure S22. The PXRD of 1D Zr-oxo MOF using TCPP as the linker.

The obtained 1D Zr-oxo MOF using TCPP as the linker shows an identical crystallographic structure to PCN-226 reported previously.[18]

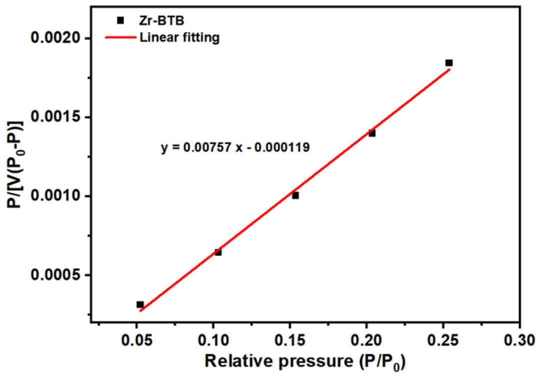

Figure S23. The fitting line of the BET surface area of Zr-BTB.

According to the BET equation, the corresponding surface area is calculated to be 584 m^2/g.

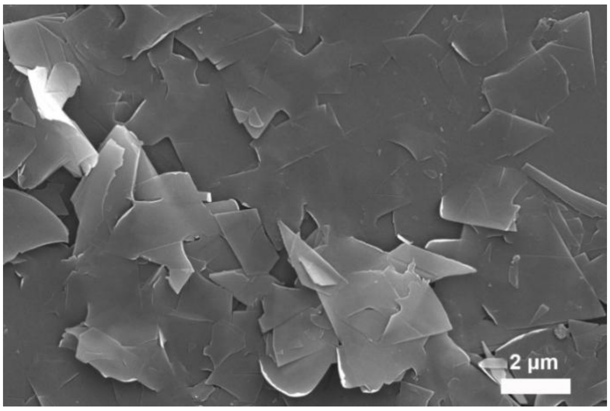

Figure S24. The SEM image of as-synthesized UMCM-309.

The resultant UMCM-309 shows nanosheet-shaped morphologies.

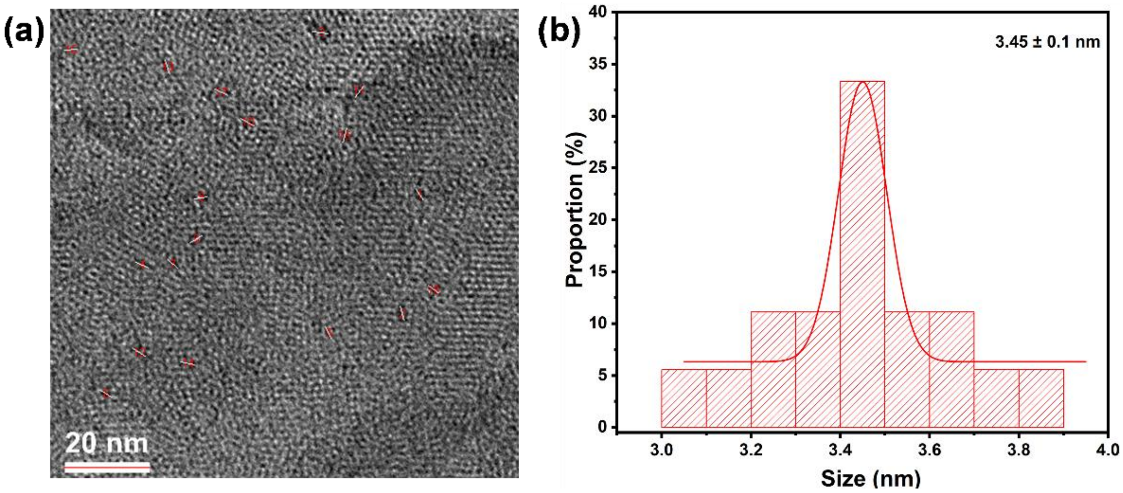

Figure S25. Particle size distribution of Pd NPs of Pd@UMCM-309.

The average particle size of Pd NPs within UMCM-309 is about 3.5 nm.

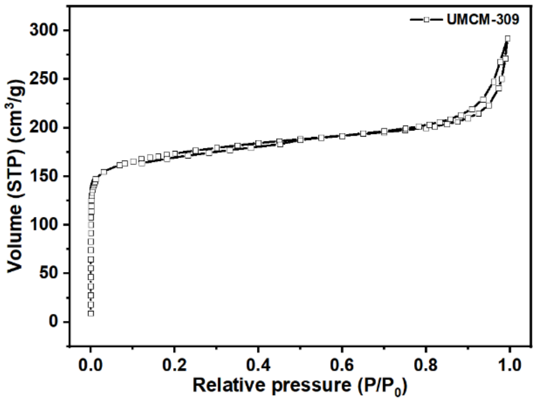

Figure S26. The N_2 adsorption-desorption isotherms of UMCM-309 probed at 77K.

As shown in Figure S26, UMCM-309 presents type I isotherm characteristic of microporosity similarly.

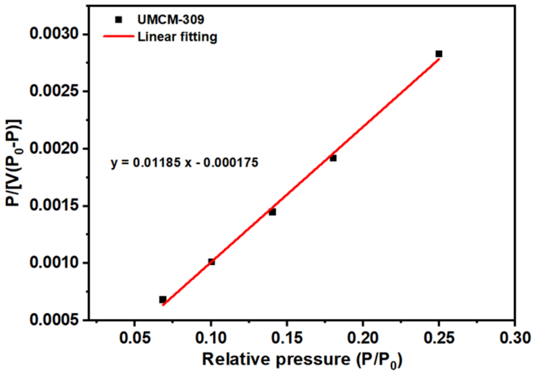

Figure S27. The fitting line of the BET surface area of UMCM-309.

According to the BET equation, the corresponding surface area is calculated to be 377 m^2/g.

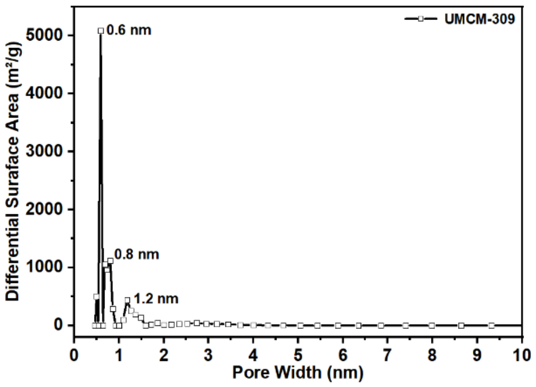

Figure S28. The pore size distribution of UMCM-309.

As shown in Figure S28, UMCM-309 presents a main pore size centered at 0.6 nm.

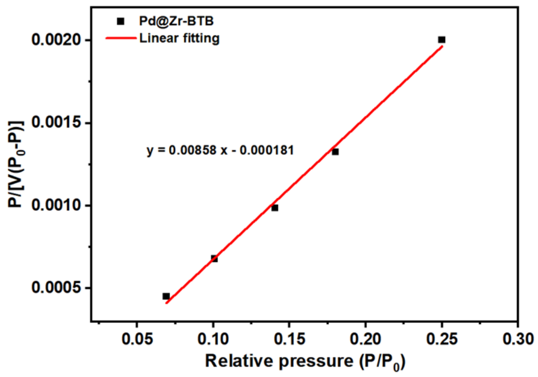

Figure S29. The fitting line of the BET surface area of Pd@Zr-BTB.

According to the BET equation, the corresponding surface area is calculated to be 524 m^2/g.

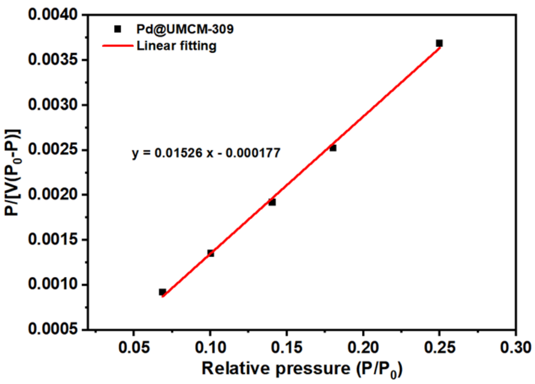

Figure S30. The fitting line of the BET surface area of Pd@UMCM-309.

According to the BET equation, the corresponding surface area is calculated to be 291 m^2/g.

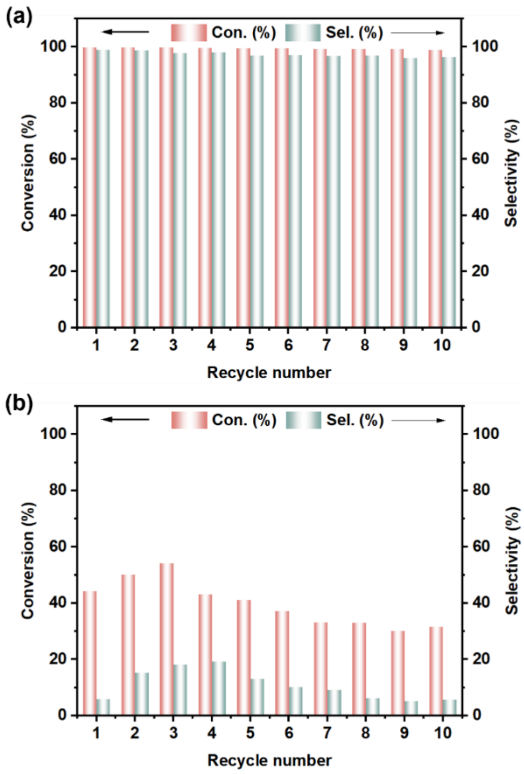

Figure S31. (a) Cyclic catalysis experiments of Pd@Zr-BTB. (b) Cyclic catalysis experiments of Pd@UMCM-309.

As shown in Figure S31, Pd@UMCM-309 presents an inferior conversion rate and poor *p*-creosol selectivity across ten successive catalysis cycles. Differently, Pd@Zr-BTB maintains above 99.0% substrate conversion ratio and over 96.0% selectivity.

The ICP-MS data on potential Pd leaching shows that there is only 0.77% Pd leaching of loaded Pd NPs after ten successive catalysis cycles.

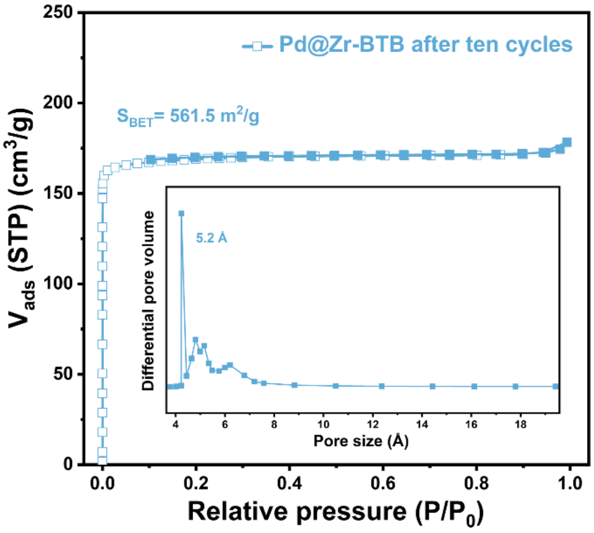

Figure S32. N_2 adsorption-desorption isotherms of Pd@Zr-BTB after ten catalytic cycles. Inset shows the pore size distribution.

As shown in Figure S32, Pd@Zr-BTB after ten catalytic cycles shows corresponding Brunauer-Emmett-Teller (BET) surface area is calculated to be 561.5 m^2/g. And the experimentally measured pore size distribution of Pd@Zr-BTB after ten catalytic cycles (inset in Figure S32) shows a primary peak centered at 5.2 Å.

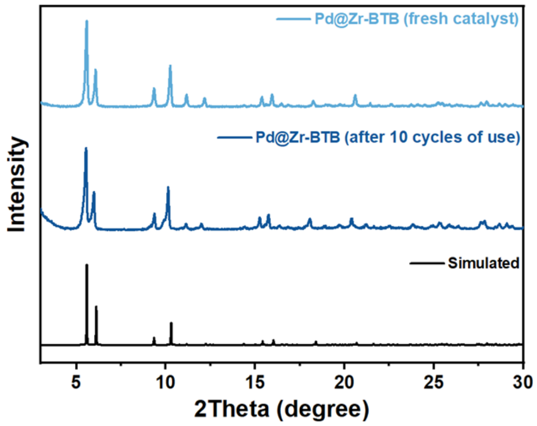

Figure S33. PXRD patterns of Pd@Zr-BTB before and after the recycle test.

As shown in Figure S33, Pd@Zr-BTB is able to maintain its crystallinity after the ten-recycle test.

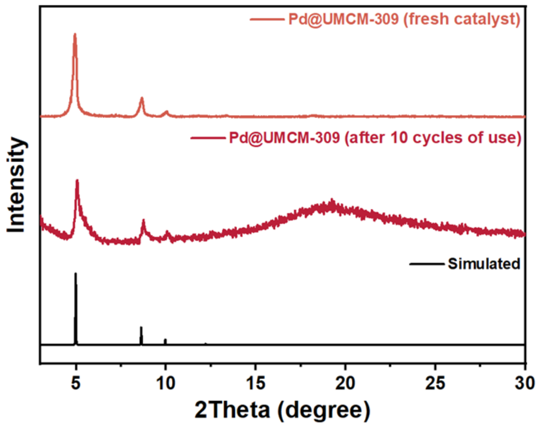

Figure S34. PXRD patterns of UMCM-309 before and after the recycle test.

As shown in Figure S34, Pd@UMCM-309 shows obviously broadened crystalline peaks after the ten-recycled test.

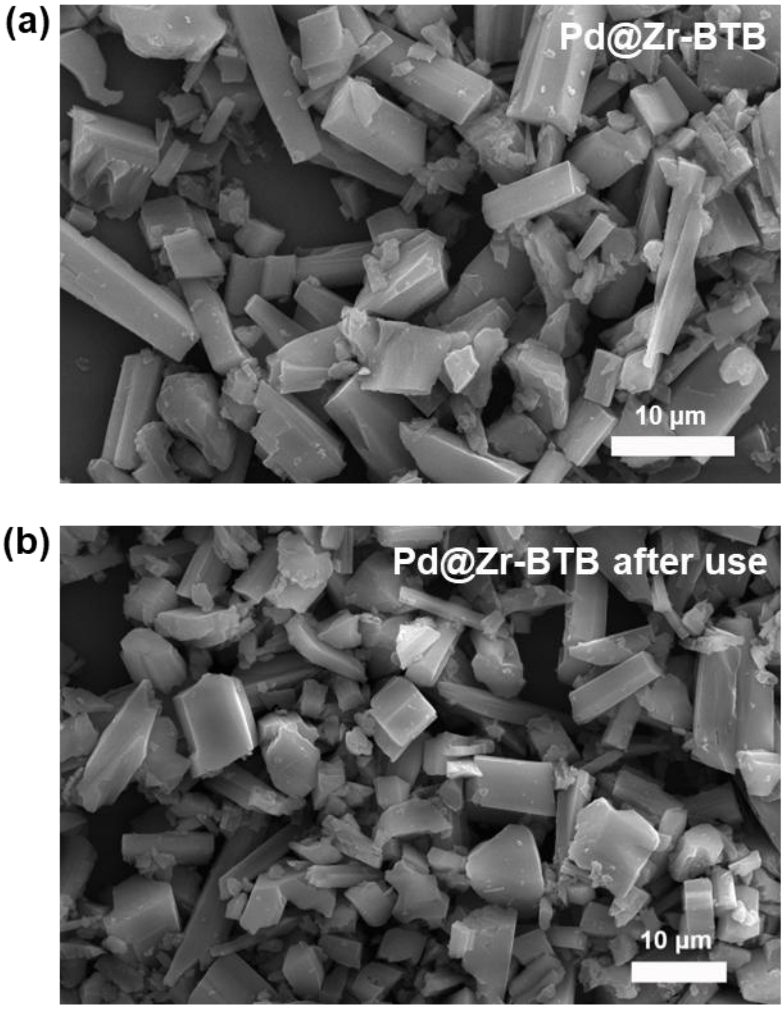

Figure S35. (a) The SEM image of Pd@Zr-BTB. (b) The SEM image of Pd@Zr-BTB after the recycling test.

As shown in Figure S35, Pd@Zr-BTB shows no obvious aggregation after the recycle test.

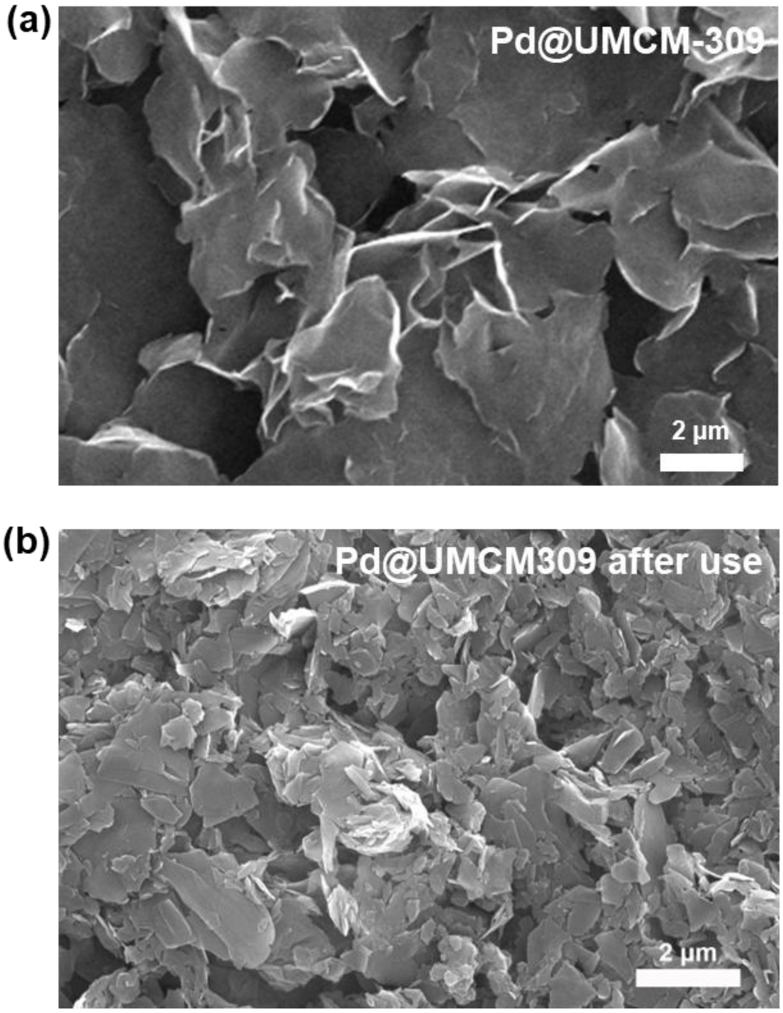

Figure S36. (a) The SEM image of Pd@UMCM-309. (b) The SEM image of Pd@UMCM-309 after the recycling test.

As shown in Figure S36, Pd@UMCM-309 appears to be fragmented and aggregated after the recycle test.

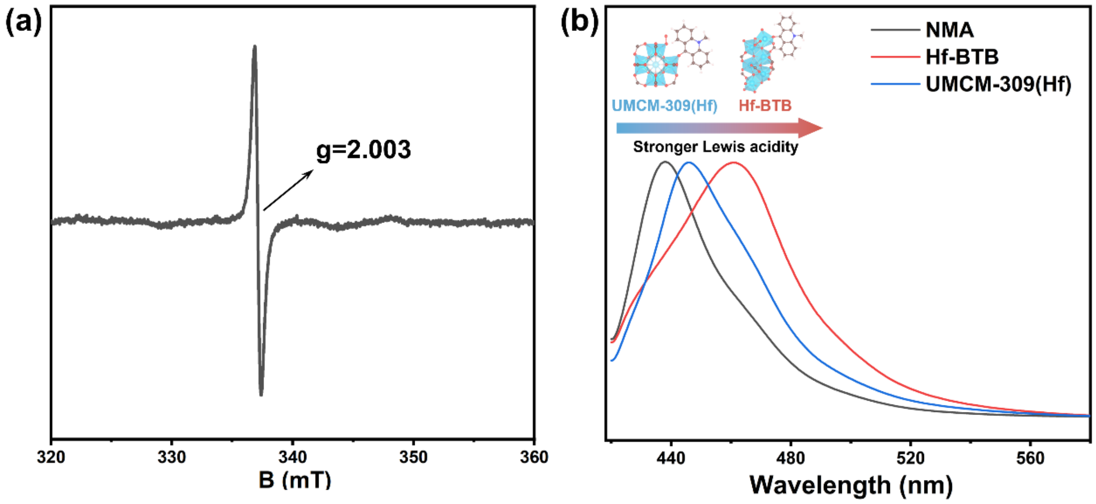

Figure S37. (a) EPR spectra of Hf-BTB; (b) Fluorescence spectra of NMA probe upon binding to Hf-BTB and UMCM-309(Hf).

As shown in Figure S37a, a symmetrical differential signal at the proportionality factor (g-factor) of 2.003 is observed for Hf-BTB. For the results of NMA-based FL (Figure S37b), the observed emission peak centered at 461.2 nm for Hf-BTB is clearly more red-shifted than the 445.8 nm observed for UMCM-309(Hf), demonstrating the stronger Lewis acidity of Hf-BTB over UMCM-309(Hf).

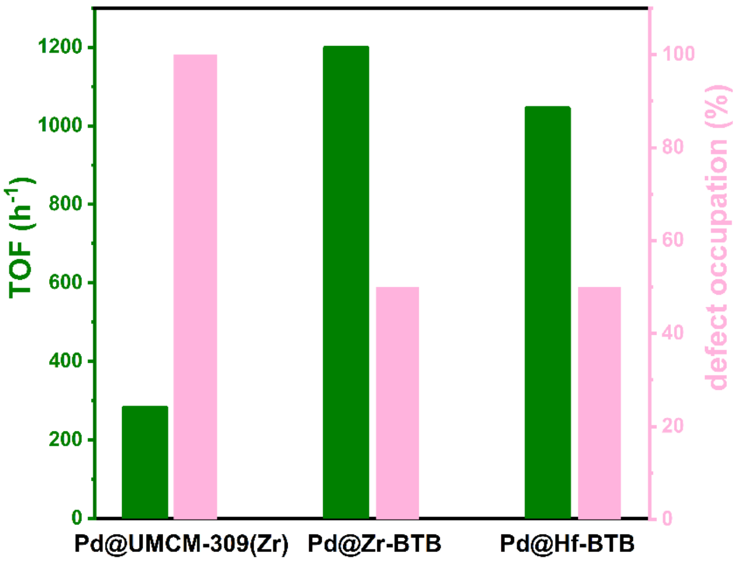

Figure S38. Data of the correlation between defect occupation and TOF per defect among Zr-BTB, Hf-BTB and UMCM-309(Zr).

Remarkably, the observed TOF sequences completely deviate the estimated defect numbers, in return highlighting the intrinsic activity discrepancy between 1D metal-oxo chain with discrete metal node.

SUPPLYMENTARY TABLES

Table S1 1D Zr-oxo MOFs structurally characterized in previous literature

MOF	1D Metal-oxo	Linker	Solvothermal Conditions	Product state	Crystallography	Ref.
MIL-140	$[ZrO]_\infty$	1,4-dicarboxybenzene	DMF	Powders	Computational modeling and PXRD refinement	Angew. Chem., Int. Ed., 2012, 51, 9267.
PCN-226	$[ZrO]_\infty$	Tetrakis(4-carboxyphenyl)porphyrin	DMF (Benzoic acid as modulator)	Powders (10 μm × 0.5 μm × 0.5 μm)	3D electron diffraction and PXRD refinement	J. Am. Chem. Soc., 2020, 142, 15386.
MIL-163	$[ZrO_8]_\infty$	5,5'(1,2,4,5-tetrazine-3,6-diyl)bis(benzene-1,2,3-triol)	DMF&H_2O	Powders	Computational modeling and PXRD refinement	Angew. Chem., Int. Ed., 2015, 54, 13297.
CAU-22	$[Zr_6O_4(OH)_4(\mu_2\text{-}OH)_2]_\infty$	2,5-pyrazinedicarboxylic acid	DMF	Powders (0.5 μm × 0.3 μm × 0.3 μm)	Computational modeling and PXRD refinement	Chem. Commun., 2016, 52, 12698.
CAU-27	$[Zr_6O_4(OH)_4]_\infty$	1,4-dicarboxybenzene	DMF	Powders (0.9 μm × 0.1 μm × 0.1 μm)	Computational modeling and PXRD refinement	Angew. Chem., Int. Ed., 2019, 58, 10995.
ZrPP-1	$[ZrO_8]_\infty$	5,10,15,20-tetrakis(3,4,5-methoxyphenyl)porphyrin	DMF&H_2O	Powders (0.3 μm × 0.3 μm × 0.1 μm)	Computational modeling and PXRD refinement	Adv. Mater., 2018, 30, 1704388.
Zr-BTB	$[Zr_3(\mu_3\text{-}O)_3]_\infty$	Benzene-1,3,5-tribenzoic acid	Acetic acid	Single crystal (95 μm × 28 μm × 28 μm)	Absolute SXRD	This work
Hf-BTB	$[Hf_3(\mu_3\text{-}O)_3]_\infty$	Benzene-1,3,5-tribenzoic acid	Acetic acid	Single crystal (115 μm × 43 μm × 43 μm)	Absolute SXRD	This work

Table S2. The summarized crystallographic information of Zr-BTB.

Empirical formula	$C_{54}H_{30}O_{15}Zr_3$
Formula weight	1192.502
CCDC	2040243
Temperature/K	193.01
Crystal system	monoclinic
Space group	C2/c
a/Å	30.7290(11)
b/Å	18.8717(7)
c/Å	11.3641(5)
α/°	90
β/°	106.701(2)
γ/°	90
Volume/Å^3	6312.1(4)
Z	4
ρ_{calc}g/cm^3	1.255
μ/mm^{-1}	2.980
F(000)	2377.3
Crystal size/mm^3	0.120 × 0.110 × 0.080
Radiation	GaKα (λ = 1.34139)
2Θ range for data collection/°	4.84 to 108.048

Index ranges	$-36 \leq h \leq 36$, $-22 \leq k \leq 22$, $-13 \leq l \leq 13$
Reflections collected	40666
Independent reflections	5808 [R_{int} = 0.0762, R_{sigma} = 0.0418]
Data/restraints/parameters	5808/0/326
Goodness-of-fit on F^2	1.010
Final R indexes [I>=2σ (I)]	R_1 = 0.0321, wR_2 = 0.0811
Final R indexes [all data]	R_1 = 0.0426, wR_2 = 0.0862
Largest diff. peak/hole / e $Å^{-3}$	1.07/-1.09

Table S3. Fractional Atomic Coordinates (×10^4) and Equivalent Isotropic Displacement Parameters (Å2 ×10^3) for Zr-BTB. U_{eq} is defined as 1/3 of the trace of the orthogonalised U_{IJ} tensor.

**Atom**	***x***	***y***	***z***	**U(eq)**
C1	5586.5(10)	1794.5(13)	6362(3)	22.0(6)
C2	5863.5(10)	2449.6(14)	6581(3)	27.6(7)
C3	6207.0(11)	2538.6(15)	6023(3)	35.5(8)
C4	6429.9(11)	3181.2(15)	6114(3)	36.8(8)
C5	6321.3(10)	3747.0(15)	6765(3)	34.0(8)
C6	5995.3(12)	3632.2(15)	7391(3)	40.0(9)
C7	5768.5(12)	2990.2(16)	7297(3)	38.2(8)
C8	6529.4(10)	4450.0(15)	6751(3)	33.8(8)
C9	6958.6(11)	4515.0(16)	6575(4)	40.1(8)
C10	7149.9(11)	5175.8(15)	6491(4)	37.5(8)
C11	6900.8(11)	5785.8(15)	6568(3)	36.8(8)
C12	6477.5(11)	5734.3(14)	6762(3)	33.4(8)
C13	6293.2(11)	5070.1(15)	6844(3)	35.1(8)
C14	6215.4(11)	6384.1(14)	6854(3)	32.2(7)
C15	6129.8(13)	6903.8(17)	5945(4)	45.4(9)
C16	5889.9(13)	7511.0(18)	6050(3)	45.2(9)
C17	5744.7(11)	7620.2(15)	7073(3)	30.7(7)
C18	5813.6(13)	7093.2(17)	7960(3)	44.6(9)

C19	6043.5(13)	6482.4(17)	7836(4)	45.6(9)
C20	5505.8(11)	8289.9(15)	7229(3)	29.8(7)
C21	7617.2(11)	5214.5(16)	6367(4)	40.2(9)
C22	7758.4(12)	4746.7(18)	5604(4)	48.2(10)
C23	8204.0(11)	4750.9(17)	5555(4)	44.2(9)
C24	8512.5(11)	5230.8(16)	6265(3)	37.5(8)
C25	8373.4(11)	5705.6(17)	7009(4)	44.0(9)
C26	7926.0(11)	5700.8(16)	7052(4)	42.7(9)
C27	8996.7(10)	5210.9(15)	6259(3)	30.8(7)
O1	5278.3(7)	1740.6(9)	6898.0(19)	26.4(5)
O2	5660.8(6)	1341.5(9)	5633.9(18)	24.5(4)
O3	5478.1(8)	8449.7(10)	8266.2(19)	34.7(5)
O4	5347.6(7)	8656.2(10)	6252.0(19)	32.0(5)
O5	9265.2(7)	5660.7(9)	6912(2)	28.4(5)
O6	9115.0(7)	4743.4(10)	5614(2)	30.3(5)
O7	5000	9681.4(12)	7500	21.8(6)
O8	5059.4(6)	9542.3(9)	4287.5(17)	21.7(4)
Zr1	5000	9203.60(15)	2500	17.17(10)
Zr2	4789.06(8)	9358.74(11)	5718.7(2)	18.44(9)

Table S4. Anisotropic displacement parameters ($Å^2 \times 10^3$) for Zr-BTB.

**Atom**	**U11**	**U22**	**U33**	**U23**	**U13**	**U12**
C1	25.4(16)	13.7(12)	31.3(16)	0.1(11)	15.2(13)	1.8(11)
C2	32.1(17)	16.8(13)	41.2(18)	-6.1(12)	22.1(15)	-3.5(12)
C3	37.5(19)	20.8(14)	59(2)	-4.9(13)	30.9(17)	-8.6(14)
C4	33.4(19)	22.2(15)	65(2)	-8.2(13)	31.0(17)	-6.9(15)
C5	31.7(18)	16.8(14)	59(2)	-3.2(12)	22.2(16)	-2.3(14)
C6	47(2)	20.3(15)	66(2)	-9.1(14)	36.8(19)	-13.1(15)
C7	44(2)	26.1(16)	58(2)	-9.9(14)	35.1(18)	-10.1(15)
C8	25.8(17)	21.5(14)	58(2)	-3.1(12)	18.7(16)	-8.8(14)
C9	28.9(18)	20.9(14)	76(3)	0.1(13)	24.6(17)	-8.1(16)
C10	26.1(17)	22.5(15)	70(2)	-3.2(12)	23.5(17)	-7.0(15)
C11	29.7(18)	17.7(14)	68(2)	-4.5(12)	22.2(17)	-4.4(15)
C12	28.1(17)	19.5(14)	57(2)	-1.3(12)	19.0(16)	-7.0(14)
C13	28.1(18)	22.8(15)	60(2)	-4.4(12)	21.1(17)	-5.5(14)
C14	33.6(18)	18.5(14)	50(2)	1.2(12)	21.1(16)	-4.0(13)
C15	61(2)	35.2(18)	52(2)	12.9(16)	35(2)	-3.0(16)
C16	64(3)	34.8(18)	45(2)	16.7(17)	29.6(19)	6.0(16)
C17	38.6(19)	19.2(14)	38.3(18)	6.7(13)	17.6(15)	-0.2(13)
C18	60(2)	33.3(17)	50(2)	15.8(17)	30.6(19)	4.8(16)
C19	60(2)	28.6(17)	55(2)	14.9(16)	27(2)	8.5(16)
C20	38.7(19)	20.9(14)	35.0(18)	7.5(13)	18.9(15)	0.2(13)

C21	28.1(18)	22.6(15)	78(3)	-3.3(13)	27.9(18)	-10.0(16)
C22	30.9(19)	33.7(18)	85(3)	-10.9(15)	24.3(19)	-26.2(18)
C23	28.2(18)	35.4(18)	75(3)	-3.9(14)	24.3(18)	-22.7(18)
C24	24.5(17)	27.8(15)	64(2)	-4.0(13)	17.8(16)	-15.4(15)
C25	27.4(18)	34.8(18)	76(3)	-10.0(13)	24.7(19)	-25.4(17)
C26	29.7(19)	30.6(17)	75(3)	-5.6(13)	26.3(19)	-19.7(16)
C27	23.0(16)	25.2(15)	48(2)	-0.9(12)	16.9(15)	-4.4(14)
O1	29.5(11)	13.9(9)	43.7(13)	-3.1(8)	23.4(10)	0.2(8)
O2	27.8(11)	19.1(9)	31.8(11)	-5.7(8)	16.9(9)	-5.8(8)
O3	51.6(14)	23.9(10)	34.4(12)	15.8(10)	21.5(11)	-0.3(9)
O4	42.7(13)	25.1(10)	35.9(12)	15.8(9)	23.9(11)	8.4(9)
O5	22.0(11)	21.3(10)	46.7(13)	-4.4(8)	17.3(10)	-11.6(9)
O6	25.5(11)	23.6(10)	46.5(13)	-1.5(8)	18.0(10)	-11.9(9)
O7	24.7(15)	14.7(12)	28.6(15)	-0	12.0(12)	0
O8	25.4(10)	12.9(8)	30.3(11)	-2.9(8)	13.6(9)	1.1(8)
Zr1	18.85(18)	10.16(15)	26.69(19)	-0	13.23(14)	0
Zr2	21.69(14)	11.07(12)	27.38(15)	-2.26(8)	14.75(11)	-1.07(9)

Table S5. Bond Lengths for Zr-BTB.

Atom	Atom	Length/Å	Atom	Atom	Length/Å
C1	C2	1.481(4)		C20	O4
C1	O1	1.269(3)		C21	C22
C1	O2	1.256(3)		C21	C26
C2	C3	1.389(4)		C22	C23
C2	C7	1.387(4)		C23	C24
C3	C4	1.382(4)		C24	C25
C4	C5	1.394(4)		C24	C27
C5	C6	1.402(4)		C25	C26
C5	C8	1.475(4)		C27	O5
C6	C7	1.386(4)		C27	O6
C8	C9	1.395(4)		O1	Zr1^{1}
C8	C13	1.397(4)		O2	Zr2^{2}
C9	C10	1.394(4)		O3	Zr2^{3}
C10	C11	1.399(4)		O4	Zr2
C10	C21	1.485(4)		O5	Zr1^{4}
C11	C12	1.384(4)		O6	Zr2^{5}
C12	C13	1.389(4)		O7	Zr1^{6}
C12	C14	1.487(4)		O7	Zr2
C14	C15	1.394(5)		O7	Zr2^{3}
C14	C19	1.378(5)		O8	Zr1

C15	C16	1.386(4)	O8	Zr2[6]
C16	C17	1.376(4)	O8	Zr2
C17	C18	1.389(4)	Zr1	Zr2[6]
C17	C20	1.498(4)	Zr1	Zr2[7]
C18	C19	1.380(4)	Zr2	Zr2[6]
C20	O3	1.243(3)		

[1]+X,1-Y,1/2+Z; [2]1-X,1-Y,1-Z; [3]1-X, +Y,3/2-Z; [4]3/2-X,3/2-Y,1-Z; [5]1/2+X, -1/2+Y, +Z; [6]1-X,2-Y,1-Z; [7]+X,2-Y, -1/2+Z

Table S6. Bond Angles for Zr-BTB.

Atom	Atom	Atom	Angle/°	Atom	Atom	Atom	Angle/°
O1	C1	C2	117.3(2)	$O5^{8}$	Zr1	$O5^{4}$	166.50(9)
O2	C1	C2	118.3(2)	$O7^{6}$	Zr1	$O1^{7}$	145.18(5)
O2	C1	O1	124.3(2)	$O7^{6}$	Zr1	$O1^{2}$	145.18(5)
C3	C2	C1	120.0(3)	$O7^{6}$	Zr1	$O5^{8}$	83.25(5)
C7	C2	C1	120.3(3)	$O7^{6}$	Zr1	$O5^{4}$	83.25(5)
C7	C2	C3	119.6(3)	O8	Zr1	$O1^{7}$	128.76(7)
C4	C3	C2	119.8(3)	O8	Zr1	$O1^{2}$	82.92(7)
C5	C4	C3	121.5(3)	$O8^{9}$	Zr1	$O1^{7}$	82.92(7)
C6	C5	C4	117.8(3)	$O8^{9}$	Zr1	$O1^{2}$	128.76(7)
C8	C5	C4	120.8(3)	O8	Zr1	$O5^{4}$	82.68(8)
C8	C5	C6	121.3(3)	$O8^{9}$	Zr1	$O5^{8}$	82.68(8)
C7	C6	C5	120.8(3)	O8	Zr1	$O5^{8}$	93.18(8)
C6	C7	C2	120.2(3)	$O8^{9}$	Zr1	$O5^{4}$	93.18(8)
C9	C8	C5	120.8(3)	$O8^{9}$	Zr1	$O7^{6}$	72.17(5)
C13	C8	C5	121.1(3)	O8	Zr1	$O7^{6}$	72.17(5)
C13	C8	C9	118.1(3)	$O8^{9}$	Zr1	O8	144.33(10)
C10	C9	C8	121.6(3)	$Zr2^{10}$	Zr1	$O1^{7}$	120.13(5)
C11	C10	C9	118.9(3)	$Zr2^{6}$	Zr1	$O1^{7}$	146.48(5)
C21	C10	C9	119.3(3)	$Zr2^{10}$	Zr1	$O1^{2}$	146.48(5)
C21	C10	C11	121.8(3)	$Zr2^{6}$	Zr1	$O1^{2}$	120.13(5)

C12	C11	C10	120.6(3)	$Zr2^{10}$	Zr1	$O5^{8}$	73.43(5)
C13	C12	C11	119.6(3)	$Zr2^{10}$	Zr1	$O5^{4}$	95.39(5)
C14	C12	C11	120.4(3)	$Zr2^{6}$	Zr1	$O5^{8}$	95.39(5)
C14	C12	C13	120.0(3)	$Zr2^{6}$	Zr1	$O5^{4}$	73.43(5)
C12	C13	C8	121.3(3)	$Zr2^{6}$	Zr1	$O7^{6}$	35.567(5)
C15	C14	C12	121.2(3)	$Zr2^{10}$	Zr1	$O7^{6}$	35.567(5)
C19	C14	C12	120.6(3)	$Zr2^{6}$	Zr1	O8	38.07(5)
C19	C14	C15	118.1(3)	$Zr2^{6}$	Zr1	$O8^{9}$	106.80(5)
C16	C15	C14	120.7(3)	$Zr2^{10}$	Zr1	O8	106.80(5)
C17	C16	C15	120.4(3)	$Zr2^{10}$	Zr1	$O8^{9}$	38.07(5)
C18	C17	C16	119.2(3)	$Zr2^{10}$	Zr1	$Zr2^{6}$	71.134(10)
C20	C17	C16	121.1(3)	$O3^{3}$	Zr2	$O2^{2}$	70.38(7)
C20	C17	C18	119.7(3)	O4	Zr2	$O2^{2}$	96.86(8)
C19	C18	C17	120.0(3)	O4	Zr2	$O3^{3}$	76.72(8)
C18	C19	C14	121.5(3)	$O6^{11}$	Zr2	$O2^{2}$	76.28(7)
O3	C20	C17	119.1(3)	$O6^{11}$	Zr2	$O3^{3}$	78.91(8)
O4	C20	C17	115.4(3)	$O6^{11}$	Zr2	O4	155.58(8)
O4	C20	O3	125.4(3)	$O7^{3}$	Zr2	$O2^{2}$	146.68(6)
C22	C21	C10	120.8(3)	$O7^{3}$	Zr2	$O3^{3}$	77.63(8)
C26	C21	C10	120.2(3)	$O7^{3}$	Zr2	O4	84.39(7)
C26	C21	C22	119.0(3)	$O7^{3}$	Zr2	$O6^{11}$	88.90(6)
C23	C22	C21	120.7(3)	$O8^{6}$	Zr2	$O2^{2}$	132.90(7)

C24	C23	C22	119.8(3)	O8	Zr2	$O2^{2}$	82.16(7)
C25	C24	C23	119.9(3)	O8	Zr2	$O3^{3}$	142.24(7)
C27	C24	C23	119.7(3)	$O8^{6}$	Zr2	$O3^{3}$	145.46(7)
C27	C24	C25	120.3(3)	O8	Zr2	O4	81.56(8)
C26	C25	C24	120.0(3)	$O8^{6}$	Zr2	O4	116.87(8)
C25	C26	C21	120.6(3)	$O8^{6}$	Zr2	$O6^{11}$	83.09(7)
O5	C27	C24	117.6(3)	O8	Zr2	$O6^{11}$	119.81(8)
O6	C27	C24	118.3(3)	O8	Zr2	$O7^{3}$	130.62(6)
O6	C27	O5	124.1(3)	$O8^{6}$	Zr2	$O7^{3}$	72.76(8)
$Zr1^{1}$	O1	C1	129.28(16)	$Zr1^{12}$	Zr2	$O2^{2}$	152.87(5)
$Zr2^{2}$	O2	C1	132.50(18)	$Zr1^{12}$	Zr2	$O3^{3}$	109.34(5)
$Zr2^{3}$	O3	C20	140.67(19)	$Zr1^{12}$	Zr2	O4	109.62(6)
Zr2	O4	C20	131.45(18)	$Zr1^{12}$	Zr2	$O6^{11}$	77.10(5)
$Zr1^{4}$	O5	C27	134.63(18)	$Zr1^{12}$	Zr2	$O7^{3}$	37.01(6)
$Zr2^{5}$	O6	C27	129.14(19)	$Zr1^{12}$	Zr2	$O8^{6}$	37.25(5)
$Zr2^{3}$	O7	$Zr1^{6}$	107.43(6)	$Zr1^{12}$	Zr2	O8	106.90(5)
Zr2	O7	$Zr1^{6}$	107.43(6)	$Zr2^{6}$	Zr2	$O2^{2}$	110.19(5)
Zr2	O7	$Zr2^{3}$	145.15(13)	$Zr2^{6}$	Zr2	$O3^{3}$	177.96(6)
Zr2	O8	Zr1	140.87(9)	$Zr2^{6}$	Zr2	O4	101.24(6)
$Zr2^{6}$	O8	Zr1	104.69(8)	$Zr2^{6}$	Zr2	$O6^{11}$	103.12(6)
$O1^{2}$	Zr1	$O1^{7}$	69.63(10)	$Zr2^{6}$	Zr2	$O7^{3}$	102.11(5)
$O5^{8}$	Zr1	$O1^{7}$	117.88(8)	$Zr2^{6}$	Zr2	O8	36.80(5)

O5^{4}	Zr1	O1^{7}	74.06(7)	Zr2^{6}	Zr2	O8^{6}	35.42(5)
O5^{4}	Zr1	O1^{2}	117.88(8)	Zr2^{6}	Zr2	Zr1^{12}	71.106(7)
O5^{8}	Zr1	O1^{2}	74.06(7)				

^{1}+X,1-Y,1/2+Z; ^{2}1-X,1-Y,1-Z; ^{3}1-X,+Y,3/2-Z; ^{4}3/2-X,3/2-Y,1-Z; ^{5}1/2+X,-1/2+Y,+Z;

^{6}1-X,2-Y,1-Z; ^{7}+X,1-Y,-1/2+Z; ^{8}-1/2+X,3/2-Y,-1/2+Z; ^{9}1-X,+Y,1/2-Z;

^{10}+X,2-Y,-1/2+Z; ^{11}-1/2+X,1/2+Y,+Z; ^{12}+X,2-Y,1/2+Z

Table S7. Torsion Angles for Zr-BTB.

A	B	C	D	Angle/°	A	B	C	D	Angle/°
C1	C2	C3	C4	172.6(3)	C11	C12	C14	C15	52.9(4)
C1	C2	C7	C6	-173.0(3)	C11	C12	C14	C19	-128.1(4)
C2	C3	C4	C5	0.5(4)	C12	C14	C15	C16	-179.2(3)
C2	C7	C6	C5	0.3(4)	C12	C14	C19	C18	177.6(3)
C3	C4	C5	C6	3.7(4)	C14	C15	C16	C17	2.1(4)
C3	C4	C5	C8	-173.8(3)	C14	C19	C18	C17	1.1(4)
C4	C5	C6	C7	-4.1(4)	C15	C16	C17	C18	-4.4(5)
C4	C5	C8	C9	-28.0(4)	C15	C16	C17	C20	176.9(3)
C4	C5	C8	C13	148.4(4)	C16	C17	C18	C19	2.9(4)
C5	C8	C9	C10	176.4(4)	C16	C17	C20	O3	-162.6(3)
C5	C8	C13	C12	-176.3(3)	C16	C17	C20	O4	16.9(4)
C8	C9	C10	C11	-0.9(5)	C17	C20	O3	Zr2[1]	176.63(15)
C8	C9	C10	C21	176.9(4)	C17	C20	O4	Zr2	151.65(18)
C8	C13	C12	C11	0.9(4)	C21	C22	C23	C24	-0.6(5)
C8	C13	C12	C14	179.5(3)	C21	C26	C25	C24	1.0(5)
C9	C10	C11	C12	1.9(4)	C22	C23	C24	C25	-0.6(5)
C9	C10	C21	C22	41.4(4)	C22	C23	C24	C27	177.0(4)
C9	C10	C21	C26	-135.9(4)	C23	C24	C25	C26	0.4(5)
C10	C11	C12	C13	-1.9(4)	C23	C24	C27	O5	179.5(3)
C10	C11	C12	C14	179.5(3)	C23	C24	C27	O6	-1.2(4)

C10	C21	C22	C23	-175.4(4)	C24	C27	O5	Zr1[2]	168.67(18)
C10	C21	C26	C25	175.3(4)	C24	C27	O6	Zr2[3]	-162.0(2)

[1]1-X, +Y,3/2-Z; [2]3/2-X,3/2-Y,1-Z; [3]1/2+X, -1/2+Y, +Z

Table S8. Hydrogen atom coordinates (Å×10^4) and isotropic displacement parameters (Å^2×10^3) for Zr-BTB.

**Atom**	***x***	***y***	***z***	**U(eq)**
H3	6288.4(11)	2158.8(15)	5579(3)	42.6(9)
H4	6662.5(11)	3238.6(15)	5723(3)	44.2(10)
H6	5929.0(12)	3998.8(15)	7886(3)	48.0(10)
H7	5547.5(12)	2920.4(16)	7723(3)	45.9(10)
H9	7124.4(11)	4099.0(16)	6511(4)	48.1(10)
H11	7023.4(11)	6239.2(15)	6487(3)	44.2(10)
H13	6000.7(11)	5037.0(15)	6966(3)	42.1(9)
H15	6237.1(13)	6841.2(17)	5247(4)	54.5(11)
H16	5825.3(13)	7853.8(18)	5411(3)	54.2(11)
H18	5702.6(13)	7153.0(17)	8652(3)	53.5(11)
H19	6084.1(13)	6121.8(17)	8442(4)	54.8(11)
H22	7547.4(12)	4421.0(18)	5112(4)	57.8(12)
H23	8298.2(11)	4426.6(17)	5037(4)	53.0(11)
H25	8583.7(11)	6035.7(17)	7491(4)	52.9(11)
H26	7830.9(11)	6033.5(16)	7555(4)	51.3(11)

Table S9. Topology of Zr-MOF linker by tritopic linkers reported in the literature.

MOF	Metal node	Linker	Connection	Topology	Ref.
UMCM-309	$Zr_6(\mu_3\text{-}O)_4(\mu_3\text{-}OH)_4$	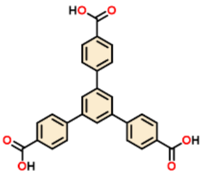 Benzene-1,3,5-tribenzoic acid	(3,6)	kgd	[19]
3D UMCM-309	$Zr_6(\mu_3\text{-}O)_4(\mu_3\text{-}OH)_4$	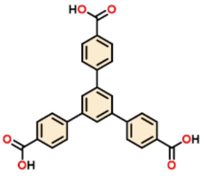 Benzene-1,3,5-tribenzoic acid	(3,6)	kgd	[19]
PCN-777	$Zr_6(\mu_3\text{-}O)_4(\mu_3\text{-}OH)_4$	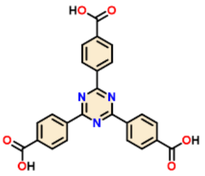 4,4',4''-s-triazine-2,4,6-triyltribenzoic acid	(3,6)	spn	[20]
MOF-808	$Zr_6(\mu_3\text{-}O)_4(\mu_3\text{-}OH)_4$	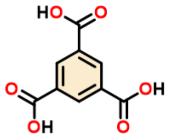 1,3,5-Benzenetricarboxylic acid	(3,6)	spn	[21]
NU-1200	$Zr_6(\mu_3\text{-}O)_4(\mu_3\text{-}OH)_4$	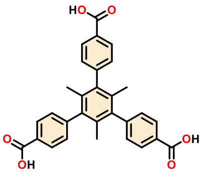 4,4',4''-(2,4,6-trimethylbenzene-1,3,5-triyl)tribenzoic acid	(3,8)	the	[22]
BUT-12	$Zr_6(\mu_3\text{-}O)_4(\mu_3\text{-}OH)_8$	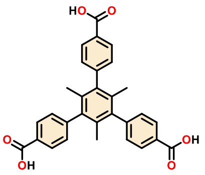	(3,8)	the	[23]

		4,4',4''-(2,4,6-trimethylbenzene-1,3,5-triyl)tribenzoic acid			
BUT-13	$Zr_6(\mu_3\text{-}O)_4(\mu_3\text{-}OH)_8$	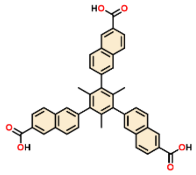 6,6',6''-(2,4,6-trimethylbenzene-1,3,5-triyl)tris(2-naphthoic acid)	(3,8)	the	[23]
MOF-1004	$Zr_6(\mu_3\text{-}O)_4(\mu_3\text{-}OH)_4$	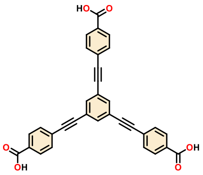 4,4',4''-(benzene-1,3,5-triyltris(ethyne-2,1-diyl))tribenzoic acid	(3,12)	sky	Journal of the American Chemical Society 2018, 140, 8958-8964.
MOF-1005	$Zr_6(\mu_3\text{-}O)_4(\mu_3\text{-}OH)_4$	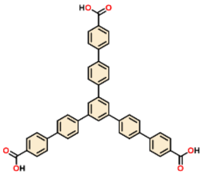 4,4',4''[benzene-1,3,5-triyl-tris(benzene-4,1-diyl)]tribenzoic acid	(3,12)	sky	Journal of the American Chemical Society 2018, 140, 8958-8964.
Zr-BTB	$[Zr_3(\mu_3\text{-}O)_3]_\infty$	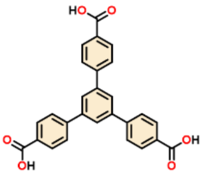 Benzene-1,3,5-tribenzoic acid	(3,7)	hyb	This work

Table S10. The summarized crystallographic information of Hf-BTB.

Empirical formula	$C_{54}H_{30}Hf_3O_{15}$
Formula weight	1454.25
CCDC No.	2330779
Temperature/K	120.00(18)
Crystal system	monoclinic
Space group	C2/a
a/Å	11.3343(3)
b/Å	18.9156(5)
c/Å	28.9350(9)
α/°	90
β/°	92.616(3)
γ/°	90
Volume/Å^3	6197.0(3)
Z	4
ρ_{calc}g/cm^3	1.559
μ/mm^{-1}	9.516
F(000)	2760.0
Crystal size/mm^3	0.14 × 0.1 × 0.08
Radiation	Cu Kα (λ = 1.54184)
2Θ range for data collection/°	5.584 to 133.2
Index ranges	$-11 \leq h \leq 13$, $-19 \leq k \leq 22$, $-34 \leq l \leq 33$
Reflections collected	15159

Independent reflections	5424 [R_{int} = 0.0636, R_{sigma} = 0.0726]
Data/restraints/parameters	5424/198/118
Goodness-of-fit on F^2	1.757
Final R indexes [I>=2σ (I)]	R_1 = 0.1490, wR_2 = 0.3413
Final R indexes [all data]	R_1 = 0.1732, wR_2 = 0.3568
Largest diff. peak/hole / e $Å^{-3}$	0.82/-0.79

Table S11. Fractional atomic coordinates ($\times 10^4$) and equivalent isotropic displacement parameters ($Å^2 \times 10^3$) for Hf-BTB. U_{eq} is defined as 1/3 of the trace of the orthogonalised U_{IJ} tensor.

**Atom**	***x***	***y***	***z***	**$U_{(eq)}$**
Hf1	7500	802.2(4)	5000	16.86(19)
Hf2	4089.3(5)	635.9(3)	4781.2(2)	17.54(1)
O1	3369(8)	8249(4)	5278(3)	17.54(1)
O2	4960(8)	8659(4)	5676(3)	17.54(1)
O3	8581(8)	5237(4)	9091(3)	17.54(1)
O4	7444(8)	4319(4)	9257(3)	17.54(1)
O5	2168(8)	1559(4)	5488(3)	17.54(1)
O6	4069(8)	1328(4)	5347(3)	17.54(1)
O7	5778(7)	458(4)	5054(3)	16.86(19)
O8	2500	303(6)	5000	17.54(1)
C1	4206(11)	8193(6)	5590(4)	17.54(1)
C2	4237(11)	7555(6)	5870(4)	17.54(1)
C3	3427(11)	6996(6)	5797(4)	17.54(1)
C4	3571(11)	6374(6)	6015(4)	17.54(1)
C5	4547(11)	6253(6)	6335(4)	17.54(1)
C6	5162(11)	7447(6)	6210(4)	17.54(1)
C7	5294(11)	6824(6)	6433(4)	17.54(1)

C8	4801(11)	5538(6)	6524(4)	17.54(1)
C9	5445(11)	5457(7)	6943(4)	17.54(1)
C10	5740(11)	4802(6)	7127(4)	17.54(1)
C11	6374(11)	4735(6)	7587(4)	17.54(1)
C12	6003(12)	4274(6)	7913(4)	17.54(1)
C13	6487(12)	4270(6)	8356(4)	17.54(1)
C14	7404(11)	4734(6)	8487(4)	17.54(1)
C15	7829(11)	5197(6)	8158(4)	17.54(1)
C16	7347(11)	5190(6)	7709(4)	17.54(1)
C17	7850(11)	4774(6)	8987(4)	17.54(1)
C18	5400(11)	4202(7)	6882(4)	17.54(1)
C19	4754(12)	4246(6)	6460(4)	17.54(1)
C20	4460(11)	4927(6)	6292(4)	17.54(1)
C21	4366(11)	3616(6)	6200(4)	17.54(1)
C22	5137(12)	3066(6)	6130(4)	17.54(1)
C23	4811(11)	2459(6)	5910(4)	17.54(1)
C24	3613(11)	2364(6)	5751(4)	17.54(1)
C25	2834(11)	2940(7)	5797(4)	17.54(1)
C26	3188(11)	3526(6)	6025(4)	17.54(1)
C27	3233(11)	1716(6)	5513(4)	17.54(1)

Table S12. Hydrogen atom coordinates (Å ×10^4) and isotropic displacement parameters ($Å^2$ ×10^3) for Hf-BTB.

**Atom**	***x***	***y***	***z***	**U(eq)**
H3	2764.53	7060.84	5588.48	21
H4	3013.23	6006.82	5955.58	21
H6	5698.68	7820.99	6281.98	21
H7	5913.63	6773.39	6663.55	21
H9	5691.17	5869.03	7109.18	21
H12	5392.9	3946.62	7830.78	21
H13	6196.38	3950.04	8577	21
H15	8448.62	5517.46	8241.61	21
H16	7663.86	5488.2	7481.37	21
H18	5610.33	3749.92	7002.92	21
H20	4010.21	4967.29	6008.05	21
H22	5934.68	3114.46	6240.62	21
H23	5378.12	2099.49	5861.7	21
H25	2052.6	2912.53	5664.46	21
H26	2630.59	3892.1	6070.39	21

Table S13. Bond lengths (Å) for Hf-BTB.

Atom	Atom	Length/Å	Atom	Atom	Length/Å
Hf1	$Hf2^{1}$	3.3392(9)	C4	C5	1.428(17)
Hf1	$Hf2^{2}$	3.3392(9)	C5	C7	1.395(17)
Hf1	$O1^{3}$	2.183(8)	C5	C8	1.482(17)
Hf1	$O1^{4}$	2.183(8)	C6	C7	1.348(17)
Hf1	$O4^{5}$	2.160(8)	C8	C9	1.395(17)
Hf1	$O4^{6}$	2.160(8)	C8	C20	1.383(17)
Hf1	O7	2.070(9)	C9	C10	1.385(17)
Hf1	$O7^{7}$	2.070(9)	C10	C11	1.488(17)
Hf1	$O8^{2}$	2.091(12)	C10	C18	1.384(17)
Hf2	$Hf2^{2}$	3.3785(12)	C11	C12	1.366(17)
Hf2	$O2^{3}$	2.195(9)	C11	C16	1.430(17)
Hf2	$O3^{8}$	2.188(8)	C12	C13	1.372(17)
Hf2	$O5^{9}$	2.364(8)	C13	C14	1.398(18)
Hf2	O6	2.097(8)	C14	C15	1.396(17)
Hf2	O7	2.064(8)	C14	C17	1.514(17)
Hf2	$O7^{2}$	2.127(8)	C15	C16	1.386(17)
Hf2	O8	2.036(4)	C18	C19	1.397(17)
O1	C1	1.282(15)	C19	C20	1.411(17)
O2	C1	1.245(15)	C19	C21	1.466(17)

O3	C17	1.233(15)	C21	C22	1.380(17)
O4	C17	1.264(15)	C21	C26	1.417(17)
O5	C27	1.242(16)	C22	C23	1.355(17)
O6	C27	1.308(15)	C23	C24	1.426(18)
C1	C2	1.455(17)	C24	C25	1.412(17)
C2	C3	1.410(17)	C24	C27	1.460(17)
C2	C6	1.419(17)	C25	C26	1.342(17)
C3	C4	1.341(17)			

[1]1/2+X,-Y,+Z; [2]1-X,-Y,1-Z; [3]1-X,1-Y,1-Z; [4]1/2+X,1-Y,+Z; [5]3/2-X,1/2-Y,3/2-Z;

[6]+X,1/2-Y,-1/2+Z; [7]3/2-X,+Y,1-Z; [8]-1/2+X,-1/2+Y,-1/2+Z; [9]1/2-X,+Y,1-Z

Table S14. Bond angles (˚) for Hf-BTB.

Atom	Atom	Atom	Angle/˚	Atom	Atom	Atom	Angle/˚
Hf2^{1}	Hf1	Hf2^{2}	70.89(3)	O8	Hf2	O5^{8}	78.6(3)
O1^{3}	Hf1	Hf2^{1}	146.9(2)	O8	Hf2	O6	84.8(3)
O1^{4}	Hf1	Hf2^{2}	146.9(2)	O8	Hf2	O7	130.5(3)
O1^{3}	Hf1	Hf2^{2}	120.1(2)	O8	Hf2	O7^{1}	71.6(4)
O1^{4}	Hf1	Hf2^{1}	120.1(2)	C1	O1	Hf1^{4}	129.4(8)
O1^{3}	Hf1	O1^{4}	69.5(4)	C1	O2	Hf2^{4}	131.3(8)
O4^{5}	Hf1	Hf2^{2}	73.5(2)	C17	O3	Hf2^{10}	127.8(8)
O4^{5}	Hf1	Hf2^{1}	96.4(2)	C17	O4	Hf1^{6}	133.8(8)
O4^{6}	Hf1	Hf2^{1}	73.5(2)	C27	O5	Hf2^{8}	140.0(8)
O4^{6}	Hf1	Hf2^{2}	96.4(2)	C27	O6	Hf2	132.2(7)
O4^{5}	Hf1	O1^{4}	74.2(3)	Hf1	O7	Hf2^{1}	105.4(4)
O4^{6}	Hf1	O1^{4}	116.5(3)	Hf2	O7	Hf1	141.3(4)
O4^{6}	Hf1	O1^{3}	74.2(3)	Hf2	O7	Hf2^{1}	107.4(4)
O4^{5}	Hf1	O1^{3}	116.5(3)	Hf2^{8}	O8	Hf1^{1}	108.0(3)
O4^{5}	Hf1	O4^{6}	167.9(4)	Hf2	O8	Hf1^{1}	108.0(3)
O7^{7}	Hf1	Hf2^{1}	106.1(2)	Hf2	O8	Hf2^{8}	144.0(6)
O7	Hf1	Hf2^{2}	106.1(2)	O1	C1	C2	117.4(11)
O7	Hf1	Hf2^{1}	37.9(2)	O2	C1	O1	124.1(11)
O7^{7}	Hf1	Hf2^{2}	37.9(2)	O2	C1	C2	118.4(11)

O7	Hf1	$O1^{3}$	129.9(3)	C3	C2	C1	122.6(11)
$O7^{7}$	Hf1	$O1^{4}$	129.9(3)	C3	C2	C6	116.9(11)
$O7^{7}$	Hf1	$O1^{3}$	82.8(3)	C6	C2	C1	120.3(11)
O7	Hf1	$O1^{4}$	82.8(3)	C4	C3	C2	121.5(12)
$O7^{7}$	Hf1	$O4^{5}$	82.9(3)	C3	C4	C5	121.4(12)
$O7^{7}$	Hf1	$O4^{6}$	93.3(3)	C4	C5	C8	121.2(11)
O7	Hf1	$O4^{6}$	82.9(3)	C7	C5	C4	116.9(11)
O7	Hf1	$O4^{5}$	93.3(3)	C7	C5	C8	121.7(11)
O7	Hf1	$O7^{7}$	143.3(5)	C7	C6	C2	121.3(11)
$O7^{7}$	Hf1	$O8^{1}$	71.7(2)	C6	C7	C5	121.8(11)
O7	Hf1	$O8^{1}$	71.7(2)	C9	C8	C5	120.4(11)
$O8^{1}$	Hf1	$Hf2^{2}$	35.446(13)	C20	C8	C5	122.5(11)
$O8^{1}$	Hf1	$Hf2^{1}$	35.446(13)	C20	C8	C9	117.1(11)
$O8^{1}$	Hf1	$O1^{3}$	145.3(2)	C10	C9	C8	122.7(11)
$O8^{1}$	Hf1	$O1^{4}$	145.3(2)	C9	C10	C11	121.2(11)
$O8^{1}$	Hf1	$O4^{5}$	83.9(2)	C9	C10	C18	118.7(11)
$O8^{1}$	Hf1	$O4^{6}$	83.9(2)	C18	C10	C11	120.1(11)
$Hf1^{1}$	Hf2	$Hf2^{1}$	71.01(2)	C12	C11	C10	121.3(11)
$O2^{4}$	Hf2	$Hf1^{1}$	153.3(2)	C12	C11	C16	118.2(11)
$O2^{4}$	Hf2	$Hf2^{1}$	110.5(2)	C16	C11	C10	120.4(11)
$O2^{4}$	Hf2	$O5^{8}$	70.0(3)	C11	C12	C13	121.7(12)

$O3^{9}$	Hf2	$Hf1^{1}$	76.7(2)	C14	C13	C12	120.7(12)
$O3^{9}$	Hf2	$Hf2^{1}$	103.2(2)	C13	C14	C15	119.1(11)
$O3^{9}$	Hf2	$O2^{4}$	77.0(3)	C13	C14	C17	120.1(11)
$O3^{9}$	Hf2	$O5^{8}$	79.9(3)	C15	C14	C17	120.6(11)
$O5^{8}$	Hf2	$Hf1^{1}$	110.0(2)	C16	C15	C14	119.9(12)
$O5^{8}$	Hf2	$Hf2^{1}$	177.0(2)	C15	C16	C11	120.3(11)
O6	Hf2	$Hf1^{1}$	109.6(2)	O3	C17	O4	126.4(11)
O6	Hf2	$Hf2^{1}$	100.4(2)	O3	C17	C14	117.4(11)
O6	Hf2	$O2^{4}$	96.6(3)	O4	C17	C14	116.2(11)
O6	Hf2	$O3^{9}$	156.4(3)	C10	C18	C19	121.4(11)
O6	Hf2	$O5^{8}$	76.6(3)	C18	C19	C21	122.1(11)
O6	Hf2	$O7^{1}$	115.8(3)	C20	C19	C18	117.5(11)
O7	Hf2	$Hf1^{1}$	107.1(2)	C20	C19	C21	120.3(11)
$O7^{1}$	Hf2	$Hf1^{1}$	36.7(2)	C8	C20	C19	122.6(11)
$O7^{1}$	Hf2	$Hf2^{1}$	35.7(2)	C22	C21	C19	120.8(11)
O7	Hf2	$Hf2^{1}$	36.9(2)	C22	C21	C26	116.7(11)
$O7^{1}$	Hf2	$O2^{4}$	134.0(3)	C26	C21	C19	122.4(11)
O7	Hf2	$O2^{4}$	81.8(3)	C23	C22	C21	123.2(12)
$O7^{1}$	Hf2	$O3^{9}$	83.2(3)	C22	C23	C24	119.6(12)
O7	Hf2	$O3^{9}$	119.6(3)	C23	C24	C25	117.6(11)
O7	Hf2	$O5^{8}$	141.3(3)	C23	C24	C27	120.9(11)

O7[1]	Hf2	O5[8]	145.9(3)	C25	C24	C27	121.3(11)
O7	Hf2	O6	81.2(3)	C26	C25	C24	120.6(12)
O7	Hf2	O7[1]	72.6(4)	C25	C26	C21	122.0(12)
O8	Hf2	Hf1[1]	36.6(3)	O5	C27	O6	124.2(11)
O8	Hf2	Hf2[1]	101.4(3)	O5	C27	C24	119.6(11)
O8	Hf2	O2[4]	147.2(3)	O6	C27	C24	116.2(11)
O8	Hf2	O3[9]	88.7(3)				

[1]1-X,-Y,1-Z; [2]1/2+X,-Y,+Z; [3]1/2+X,1-Y,+Z; [4]1-X,1-Y,1-Z; [5]+X,1/2-Y,-1/2+Z; [6]3/2-X,1/2-Y,3/2-Z; [7]3/2-X,+Y,1-Z; [8]1/2-X,+Y,1-Z; [9]-1/2+X,-1/2+Y,-1/2+Z; [10]1/2+X,1/2+Y,1/2+Z

Table S15. Torsion Angles for Hf-BTB.

A	B	C	D	Angle/°	A	B	C	D	Angle/°
$Hf1^{1}$	O1	C1	O2	-27.1(18)	C10	C11	C16	C15	170.8(12)
$Hf1^{1}$	O1	C1	C2	150.2(9)	C10	C18	C19	C20	0(2)
$Hf1^{2}$	O4	C17	O3	13(2)	C10	C18	C19	C21	-178.7(12)
$Hf1^{2}$	O4	C17	C14	-168.5(8)	C11	C10	C18	C19	176.9(12)
$Hf2^{1}$	O2	C1	O1	-39.9(18)	C11	C12	C13	C14	-1(2)
$Hf2^{1}$	O2	C1	C2	142.9(9)	C12	C11	C16	C15	-5.1(18)
$Hf2^{3}$	O3	C17	O4	-18.1(19)	C12	C13	C14	C15	-0.5(19)
$Hf2^{3}$	O3	C17	C14	163.2(8)	C12	C13	C14	C17	174.0(12)
$Hf2^{4}$	O5	C27	O6	3(2)	C13	C14	C15	C16	-0.4(19)
$Hf2^{4}$	O5	C27	C24	-175.7(8)	C13	C14	C17	O3	-173.4(12)
Hf2	O6	C27	O5	30.5(17)	C13	C14	C17	O4	7.8(18)
Hf2	O6	C27	C24	-151.0(8)	C14	C15	C16	C11	3.2(19)
O1	C1	C2	C3	3.1(19)	C15	C14	C17	O3	1.1(18)
O1	C1	C2	C6	177.8(11)	C15	C14	C17	O4	-177.7(12)
O2	C1	C2	C3	-179.5(12)	C16	C11	C12	C13	4.2(19)
O2	C1	C2	C6	-4.7(18)	C17	C14	C15	C16	-175.0(12)
C1	C2	C3	C4	170.5(12)	C18	C10	C11	C12	-45.6(19)
C1	C2	C6	C7	-171.5(12)	C18	C10	C11	C16	138.6(13)
C2	C3	C4	C5	0.6(19)	C18	C19	C20	C8	1(2)

C2	C6	C7	C5	1(2)	C18	C19	C21	C22	-46.7(18)
C3	C2	C6	C7	3.5(19)	C18	C19	C21	C26	130.9(14)
C3	C4	C5	C7	4.2(18)	C19	C21	C22	C23	177.0(12)
C3	C4	C5	C8	-171.1(12)	C19	C21	C26	C25	-178.4(12)
C4	C5	C7	C6	-5.1(19)	C20	C8	C9	C10	0(2)
C4	C5	C8	C9	-156.8(12)	C20	C19	C21	C22	134.6(13)
C4	C5	C8	C20	25.7(19)	C20	C19	C21	C26	-47.8(18)
C5	C8	C9	C10	-177.8(12)	C21	C19	C20	C8	179.8(12)
C5	C8	C20	C19	176.6(12)	C21	C22	C23	C24	-1.7(19)
C6	C2	C3	C4	-4.5(19)	C22	C21	C26	C25	-0.8(18)
C7	C5	C8	C9	28.1(19)	C22	C23	C24	C25	5.3(18)
C7	C5	C8	C20	-149.4(13)	C22	C23	C24	C27	-179.9(11)
C8	C5	C7	C6	170.2(12)	C23	C24	C25	C26	-6.8(18)
C8	C9	C10	C11	-176.8(12)	C23	C24	C27	O5	160.9(11)
C8	C9	C10	C18	1(2)	C23	C24	C27	O6	-17.7(17)
C9	C8	C20	C19	-1(2)	C24	C25	C26	C21	4.6(19)
C9	C10	C11	C12	132.3(14)	C25	C24	C27	O5	-24.6(18)
C9	C10	C11	C16	-43.5(18)	C25	C24	C27	O6	156.8(11)
C9	C10	C18	C19	-1(2)	C26	C21	C22	C23	-0.7(18)
C10	C11	C12	C13	-171.7(12)	C27	C24	C25	C26	178.5(12)

[1]1-X,1-Y,1-Z; [2]3/2-X,1/2-Y,3/2-Z; [3]1/2+X,1/2+Y,1/2+Z; [4]1/2-X,+Y,1-Z

Table S16. Hydrogen atom coordinates (Å ×10^4) and isotropic displacement parameters (Å^2 ×10^3) for Hf-BTB.

**Atom**	***x***	***y***	***z***	**U(eq)**
H3	2764.53	7060.84	5588.48	21
H4	3013.23	6006.82	5955.58	21
H6	5698.68	7820.99	6281.98	21
H7	5913.63	6773.39	6663.55	21
H9	5691.17	5869.03	7109.18	21
H12	5392.9	3946.62	7830.78	21
H13	6196.38	3950.04	8577	21
H15	8448.62	5517.46	8241.61	21
H16	7663.86	5488.2	7481.37	21
H18	5610.33	3749.92	7002.92	21
H20	4010.21	4967.29	6008.05	21
H22	5934.68	3114.46	6240.62	21
H23	5378.12	2099.49	5861.7	21
H25	2052.6	2912.53	5664.46	21
H26	2630.59	3892.1	6070.39	21

Table S17. Refined crystallographic data for isoretical Ce-BTB.

Sample	Ce-BTB
Empirical formula	$C_{12}H_{18}N_2O_6Ce$
Formula weight	351.65
Crystal system	Monoclinic
Space group	*C*2/*c*
a/Å	32.0760 ± 0.0118
b/Å	18.6347 ± 0.0075
c/Å	12.3095 ± 0.0058
α/ °	90
β/ °	102.7874 ± 0.0168
γ/ °	90
Adopted Refinement	Pawley Refinement
Final R_{wp}	2.93%
Final R_{wp} (without background)	3.63%
Final R_p	2.31%

Table S18. Comparison of thermal decomposition temperatures of Zr(Hf)-BTB with MOF reported in the literature.

MOF	Formula	Metal node	Linker	Atmosphere	$T_d(^oC)$	Ref.
MIL-140A	ZrO(BDC)	$Zr(\mu_3\text{-}O)_3O_4$	BDC[a]	N_2	514	Microporous and Mesoporous Materials 2020, 296, 109998.
UiO-66	$Zr_6O_4(OH)_4(BDC)_6$	$Zr_6(\mu_3\text{-}O)_4(\mu_3\text{-}OH)_4$	BDC[a]	N_2	517	Microporous and Mesoporous Materials 2020, 296, 109998.
UiO-66	$Hf_6O_4(OH)_4(BDC)_6$	$Hf_6(\mu_3\text{-}O)_4(\mu_3\text{-}OH)_4$	BDC[a]	Air	526	Journal of Materials Chemistry A 2019, 7, 7459-7469.
UiO-67	$Zr_6O_4(OH)_4(BPDC)_6$	$Zr_6(\mu_3\text{-}O)_4(\mu_3\text{-}OH)_4$	BPDC[b]	N_2	540	Journal of the American Chemical Society 2008, 130, 13850-13851.
UiO-67	$Hf_6O_4(OH)_4(BPDC)_6$	$Hf_6(\mu_3\text{-}O)_4(\mu_3\text{-}OH)_4$	BPDC[b]	Air	510	Journal of the American Chemical Society 2017, 139, 5397-5404.
UiO-68	$Zr_6O_4(OH)_4(TPDC)_6$	$Zr_6(\mu_3\text{-}O)_4(\mu_3\text{-}OH)_4$	TPDC[c]	Air	525	Nature communications 2016, 7, 1-11.
MOF-801	$Zr_6O_8H_4(Fumarate)_6$	$Zr_6O_8H_4(RCOO)_{12}$	Fumarate	Air	275	Microporous and Mesoporous Materials 2012, 152, 64-70.
Zr-BPYDC	$Zr_{12}O_8(OH)_{14}(BPYDC)_9$	$Zr_{12}(\mu_3\text{-}O)_8(\mu_3\text{-}OH)_8(\mu_2\text{-}OH)_6$	BPYDC[d]	Air	435	Inorganic chemistry 2017, 56, 8128-8134.
DUT-67	$Zr_6(\mu_3\text{-}O)_6(\mu_3\text{-}OH)_2(TDC)_4(AcO)_2$	$Zr_6(\mu_3\text{-}O)_6(\mu_3\text{-}OH)_2$	TDC[e]	Air	360	Crystal growth and design 2013, 13, 1231-1237.

DUT-67	$Hf_6(\mu_3\text{-}O)_6(\mu_3\text{-}OH)_2(TDC)_4(AcO)_2$	$Hf_6(\mu_3\text{-}O)_6(\mu_3\text{-}OH)_2$	TDC[e]	Air	390	Crystal growth and design 2013, 13, 1231-1237.
DUT-69	$Zr_6(\mu_3\text{-}O)_4\text{-}(\mu_3\text{-}OH)_4(TDC)_5(AcO)_2$	$Zr_6(\mu_3\text{-}O)_4(\mu_3\text{-}OH)_4$	TDC[e]	Air	370	Crystal growth and design 2013, 13, 1231-1237.
EHU-30	$Zr_6(\mu_3\text{-}O)_4(\mu_3\text{-}OH)_4(\mu_4\text{-}BDC)_6$	$Zr_6(\mu_3\text{-}O)_4(\mu_3\text{-}OH)_4$	BDC[a]	N_2	520	ACS Materials Letters 2020, 2, 499-504.
UMCM-309	$[Zr_6(\mu_3\text{-}O)_4(\mu_3\text{-}OH)_4(BTB)_2(OH)_6(H_2O)_3]_{0.5}(BTB)$	$Zr_6(\mu_3\text{-}O)_4(\mu_3\text{-}OH)_4$	BTB[f]	N_2	450	[19]
2D Hf-BTB	$Hf_6(\mu_3\text{-}O)_4(\mu_3\text{-}OH)_4\text{-}(HCO_2)_6(BTB)_2$	$Hf_6(\mu_3\text{-}O)_4(\mu_3\text{-}OH)_4$	BTB[f]	Air	~500	Angewandte International Edition Chemie 2016, 55, 4962-4966
3D UMCM-309	$Zr_6(\mu_3\text{-}O)_4(\mu_3\text{-}OH)_4(OH)_6(H_2O)_6(BTB)_2$ 6DMF H_2O	$Zr_6(\mu_3\text{-}O)_4(\mu_3\text{-}OH)_4$	BTB[f]	N_2	280	[19]
PCN-777	$Zr_6O_4(OH)_{10}(H_2O)_6(TATB)_2$	$Zr_6(\mu_3\text{-}O)_4(\mu_3\text{-}OH)_4$	TATB[g]	N_2	500	[20]
MOF-808	$Zr_6O_4(OH)_4(BTC)_2(HCOO)_6$	$Zr_6(\mu_3\text{-}O)_4(\mu_3\text{-}OH)_4$	BTC[h]	Air	500	[21]
Zr-BTBP	$Zr_3(H_3btbp)_4\cdot 15H_2O$	ZrO_6	BTBP[i]	N_2	400	Chemical Communications 2014, 50, 5737-5740.
UPG-1	$Zr(H_4ttbmp)_2\cdot 10H_2O$	ZrO_6	TTBMP[j]	Air	450	Chemical Communications 2014, 50, 14831-14834.
PCN-221	$Zr_8(\mu_4\text{-}O)_6(OH)_8(TCPP)_3$	$Zr_8(\mu_4\text{-}O)_6$	TCPP[k]	N_2	390	Inorganic chemistry 2013, 52, 12661-12667.
PCN-222	$Zr_6(\mu_3\text{-}OH)_8(OH)_8(TCPP)_2$	$Zr_6(\mu_3\text{-}OH)_8$	TCPP[k]	N_2	370	Angewandte Chemie International Edition 2012, 51, 10307.

PCN-223	$Zr_6(\mu_3\text{-}O)_4(\mu_3\text{-}OH)_4(TCPP)_3$	$Zr_6(\mu_3\text{-}O)_4(\mu_3\text{-}OH)_4$	TCPP[k]	N_2	360	Journal of the American Chemical Society 2014, 136, 17714-17717.
PCN-224	$Zr_6(\mu_3\text{-}O)_4(\mu_3\text{-}OH)_4(OH)_6(H_2O)_6(TCPP)_{1.5}$	$Zr_6(\mu_3\text{-}O)_4(\mu_3\text{-}OH)_4$	TCPP[k]	N_2	400	Journal of the American Chemical Society 2013, 135, 17105-17110.
PCN-225	$Zr_6(\mu_3\text{-}O)_4(\mu_3\text{-}OH)_4(OH)_4(H_2O)_4(TCPP)_2$	$Zr_6(\mu_3\text{-}O)_4(\mu_3\text{-}OH)_4$	TCPP[k]	N_2	350	Journal of the American Chemical Society 2013, 135, 13934-13938.
NU-902	$Zr_6(\mu_3\text{-}O)_4(\mu_3\text{-}OH)_4(\text{-}OH)_4(\text{-}OH_2)_4(TCPP)_2$	$Zr_6(\mu\text{-}O)_4(\mu\text{-}OH)_4$	TCPP[k]	N_2	480	Journal of the American Chemical Society 2016, 138, 14449-14457.
NU-1000	$Zr_6(\mu_3\text{–}OH)_8(OH)_8(TBAPy)_2$	$Zr_6(\mu_3\text{-}OH)_4(\mu_3\text{-}O)_4(OH)_4(OH_2)_4$	TBAPy[l]	N_2	500	Journal of the American Chemical Society 2013, 135, 10294-10297.
NU-1000	$Zr_6(\mu_3\text{–}OH)_8(OH)_8(TBAPy)_2$	$Hf_6(\mu_3\text{-}OH)_4(\mu_3\text{-}O)_4(OH)_4(OH_2)_4$	TBAPy[l]	N_2	500	Journal of the American Chemical Society 2014, 136, 45, 15861–15864
NU-901	$Zr_6(\mu_3\text{-}OH)_8(OH)_{8\text{-}2x}(Bz)_x(TBAPy)_2$	$Zr_6(\mu_3\text{-}OH)_4(\mu_3\text{-}O)_4(OH)_4(OH_2)_4$	TBAPy[l]	N_2	470	[16]
NU-1008	$Zr_6(\mu\text{-}O)_4(\mu\text{-}OH)_4(HCOO)(H_2O)_3(OH)_3(TCPB)_2$	$Zr_6(\mu\text{-}O)_4(\mu\text{-}OH)_4$	$TCPB\text{-}Br_2$[m]	N_2	490	Journal of the American Chemical Society 2020, 142, 21428-21438.
NU-906	$Zr_6(\mu\text{-}O)_4(\mu\text{-}OH)_4(OH)_4(TCPB\text{-}Br_2)_2$	$Zr_6(\mu\text{-}O)_4(\mu\text{-}OH)_4$	$TCPB\text{-}Br_2$[m]	N_2	480	Journal of the American Chemical Society 2020, 142, 21428-21438.
NU-500	$Zr_6(\mu\text{-}O)_4(\mu\text{-}OH)_4(HCOO)_4(OH)_{3.5}(H_2O)_{3.5}(TCPB\text{-}Br_2)(H_2TCPB\text{-}Br_2)_{0.25}$	$Zr_6(\mu\text{-}O)_4(\mu\text{-}OH)_4$	$TCPB\text{-}Br_2$[m]	N_2	500	Journal of the American Chemical Society 2020, 142, 21428-21438.

NU-600	$Zr_6(\mu\text{-}O)4(\mu\text{-}OH)_4(CH_3COO)_6(TCPB\text{-}Br_2)_{1.5}$	$Zr_6(\mu\text{-}O)_4(\mu\text{-}OH)_4$	$TCPB\text{-}Br_2$ [m]	N_2	470	Journal of the American Chemical Society 2020, 142, 21428-21438.
MOF-892	$Zr_6O_4(OH)_4(H_2CPB)_{1.5}(CH_3CO_2)_6$	$Zr_6O_4(OH)_4(CH_3CO_2)_6(\text{-}CO_2)_6$	CPB[n]	Air	410	ACS applied materials & interfaces 2018, 10, 733-744.
MOF-893	$Zr_6O_4(OH)_5(H_2CPB)_2(CH_3CO_2)_3(H_2O)_4$	$Zr_6O_4(OH)_5(CH_3CO_2)_3(\text{-}CO_2)_8$	CPB[n]	Air	420	ACS applied materials & interfaces 2018, 10, 733-744.
Zr-BTB	$Zr_3(\mu_3\text{-}O)_3(BTB)_2$	$[Zr_3(\mu_3\text{-}O)_3]_\infty$	BTB[f]	N_2	550	This work
Hf-BTB	$Hf_3(\mu_3\text{-}O)_3(BTB)_2$	$[Hf_3(\mu_3\text{-}O)_3]_\infty$	BTB[f]	N_2	542	This work

[a] BDC = 1,4-dicarboxybenzene
[b] BPDC = biphenyldicarboxylic acid
[c] TPDC = triphenyldicarboxylic acid
[d] BPYDC = bipyridinedicarboxylic acid
[e] TDC= 2,5thiophenedicarboxylic acid
[f] BTB = benzene-1,3,5-tribenzoic acid
[g] TATB= 4,4',4''-s-triazine-2,4,6-triyltribenzoic acid
[h] BTC = 1,3,5-benzenetricarboxylic acid
[i] BTBP = 1,3,5-tris(4-phosphonophenyl)benzene
[j] TTBMP = 2,4,6-tris(4-(phosphonomethyl)phenyl)-1,3,5-triazine
[k] TCPP = 4,4',4''-(2,4,6-trimethylbenzene-1,3,5-triyl)tribenzoic acid
[l] TBAPy = 4,4',4'',4'''-(1,9-dihydropyrene-1,3,6,8-tetrayl)tetrabenzoic acid
[m] $TCPB\text{-}Br_2$ = 1,4-dibromo-2,3,5,6-tetrakis(4-carboxyphenyl)benzene
[n] CPB = 1′,2′,3′,4′,5′,6′-hexakis(4-carboxyphenyl)-benzene

Table S19. Performance comparison of Pd@Zr(Hf)-BTB with catalysts reported in the literature.

Entry	Catalyst	Condition		Result		TOF (h^{-1})	Ref.
		Temp. (℃)	Press. (MPa)	Conv. (%)	Sel. (%)		
1	Pd/NH_2-MIL-53-d	120	0.25	100	100	195	Chemical Engineering Journal 2023, 453, 139711.
2	Pd/NUS-SO_3H	150	2.0	>99	95.2	31	Angewandte Chemie International Edition 2022, 134, e202205453.
3	Pt-Co/MWCNTs	160	1.0	100	92.3	25	Green Chemistry 2018, 20, 2894-2902.
4	Pd/Ru@GO	25	1.0	100	92.3	56	Green Chemistry 2020, 22, 2018-2027.
5	Pd/PCE	60	2.0	>99	94.4	30	Green Chemistry 2022, 24, 1570-1577.
6	Pd@HPC-DCD	60	0.15	100	100	22	Carbon 2020, 159, 451-460.
7	Pd/Al_2O_3	50	1.7	>99	90	180	ACS Catalysis 2018, 8, 11165-11173.
8	Co_1@NC-(SBA)	140	1.0	100	99.2	10	ACS Catalysis 2020, 10, 8672-8682.
9	Ru/Nb_2O_5	250	0.5	99.9	81.2	5	Nature Communications 2017, 8, 16104.
10	Pd/Py-COF	40	1.0	>99	>99	40	Nature Communications 2022, 13, 1770.
11	Pd/d-Zr-ODB-hz	70	1.0	>99.9	>99.9	250	[24]
12	Pd/HPC-NH_2	30	HCOOH[b]	100	99.3	153	Chem 2021, 7, 3069-3084.
13	Pd@$CN_{0.132}$	150	1.0	98	100	163	Journal of the American Chemical Society 2012, 134, 16987-16990.
14	Ru_1/mpg-C_3N_4	160	4	100	100	330	[25]
15	Ru/CeO_2-S	200	1.0	99.9	99.9	281	Journal of the American Chemical Society 2022, 144, 20834-20846.
16	Ru/TiO_2	220	0.1	97.4	98.4	52	[26]

17	SiO_2@Ni@NC	25	2	99.8	>99.9[a]	20.7	Advanced Science 2024, 2309303.
18	Ni SAs-N@LC	180	HCOOH[b]	98.9	97.3	2.8	Chemical Engineering Journal 2024, 496, 154315.
19	Ni_2P@C-3	180	0.1	>99.9	95.0	1.9	Chemical Engineering Journal 2024, 489, 151367.
20	PdAu/g-C_3N_4	25	0.1	99	99	89.9	Green Chemistry 2022, 24, 1096-1102.
21	Pd/PAN–Tan	50	2	99	99[a]	264	Green Chemistry 2024, 26, 5178-5186.
22	Pd/C vin $ZnCl_2$ CO_2	100	3	100	92	157	Applied Catalysis B: Environmental 2020, 268, 118425.
23	Pd/BC	60	2	>99	92.8	60	ACS Sustainable Chemistry & Engineering 2022, 10, 7277-7287.
24	12CuO/Al_2O_3	500	0.5	100	99.2	14.4	Chemical Engineering Journal 2024, 487, 150428.
25	Ru(0.5)(300)/HZSM -5	200	5	Yield: ~80%		~323.2	Journal of Hazardous Materials 2022, 423, 126525
26	Ni(10%)/MMT	180	IPA[c]	>99	95.2	3.9	ChemCatChem 2024,16, e202301636
27	Pd@GO	25	1.0	90	84.5	243.4	Green Chemistry 2020, 22, 2018-2027.
28	Ru@GO	25	1.0	97	94.6	216.8	Green Chemistry 2020, 22, 2018-2027.
**29**	**Pd@Zr-BTB**	**60**	**0.5**	**>99.9**	**99.0**	**639.9**	**This work**
**30**	**Pd@Hf-BTB**	**60**	**0.5**	**>99.9**	**97.9**	**632.8**	**This work**

[a] Selectivity of vanillyl alcohol

[b] Using HCOOH as the hydrogen source

[c] Using Isopropanol as the hydrogen source

Table S20. The kinetic TOF summery based on low conversion ratios.

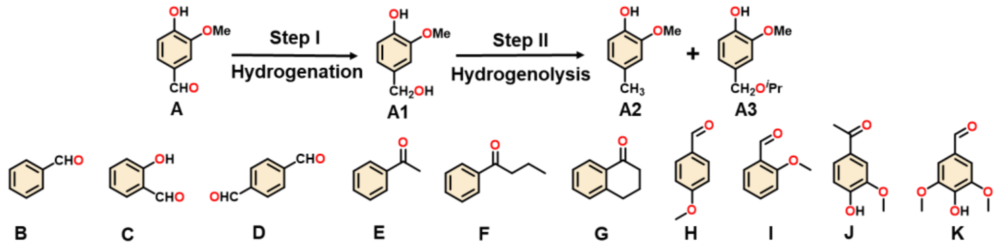

Entry	Catalyst	Substrate	T (°C)	H_2 pressure (MPa)	Time (min)	Conversion %	TOF (h^{-1})
1	Pd@UMCM-309	A	60	0.5	15	14.7	282.5
2	Pd@Zr-BTB	A	60	0.5	5	20.8	1199.1
3	Pd@Hf-BTB	A	60	0.5	5	18.1	1044.9
4	Pd/C	A	60	0.5	5	20.0	1153.0
5	Pd/ZrO_2	A	60	0.5	5	19.5	1124.1
6	Pd@Zr-BTB	B	60	0.5	5	19.9	1147.2
7	Pd@Zr-BTB	C	60	0.5	5	17.5	1008.9
8	Pd@Zr-BTB	D	60	0.5	10	25.0	720.6
9	Pd@Zr-BTB	E	60	0.5	10	21.9	631.3
10	Pd@Zr-BTB	F	60	0.5	10	24.6	711.0
11	Pd@Zr-BTB	G	60	0.5	10	24.1	695.4
12	Pd@Zr-BTB	H	60	0.5	10	24.5	706.2
13	Pd@Zr-BTB	I	60	0.5	10	20.0	576.5
14	Pd@Zr-BTB	J	60	0.5	10	14.6	420.8
15	Pd@Zr-BTB	K	60	0.5	10	22.0	634.1

Table S21. ICP analysis of reaction supernatants.

**Element**	**Element content (ug/L)**	**Total Pd NPs (ug/L)**	**Occupation ratio**
Pd	79.6029	10338.0390	0.77%

As ICP result shown in Table S21, the potential Pd leaching is only 0.77% Pd in relation to loaded Pd NPs, confirming the unchanged composition of Pd@Zr-BTB catalyst after reaction.

REFERENCES

1. Fu G, Bueken B, De Vos D. Zr-metal-organic framework catalysts for oxidative desulfurization and their improvement by postsynthetic ligand exchange. *Small Methods* 2018;2:1800203.
2. Aijaz A, Karkamkar A, Choi YJ, et al. Immobilizing highly catalytically active Pt nanoparticles inside the pores of metal-organic framework: a double solvents approach. *J Am Chem Soc* 2012;134:13926-13929.
3. Ji P, Drake T, Murakami A, Oliveres P, Skone JH, Lin W. Tuning Lewis acidity of metal-organic frameworks via perfluorination of bridging ligands: spectroscopic, theoretical, and catalytic studies. *J Am Chem Soc* 2018;140:10553-10561.
4. Willems TF, Rycroft CH, Kazi M, Meza JC, Haranczyk M. Algorithms and tools for high-throughput geometry-based analysis of crystalline porous materials. *Microporous and Mesoporous Mater* 2012;149:134-141.
5. Perdew JP, Yue W. Accurate and simple density functional for the electronic exchange energy: generalized gradient approximation. *Phys Rev B* 1986;33:8800.
6. Li K, Li N, Yan N, et al. Adsorption of small hydrocarbons on pristine, N-doped and vacancy graphene by DFT study. *Appl Surf Sci* 2020;515:146028.
7. Ernzerhof M, Perdew JP, Burke K. Coupling-constant dependence of atomization energies. *Int J Quantum Chem* 1997;64:285-295.
8. Ernzerhof M, Scuseria GE. Assessment of the Perdew–Burke–Ernzerhof exchange-correlation functional. *J Chem Phys* 1999;110:5029-5036.
9. Kresse G, Furthmüller J. Efficient iterative schemes for ab initio total-energy calculations using a plane-wave basis set. *Phys Rev B* 1996;54:11169.
10. Kresse G, Hafner J. Ab initio molecular-dynamics simulation of the liquid-metal–amorphous-semiconductor transition in germanium. *Phys Rev B* 1994;49:14251.
11. Kresse G, Joubert D. From ultrasoft pseudopotentials to the projector augmented-wave method. *Phys Rev B* 1999;59:1758.
12. Grimme S, Antony J, Ehrlich S, Krieg H. A consistent and accurate ab initio parametrization of density functional dispersion correction (DFT-D) for the 94 elements H-Pu. *J Chem Phys* 2010;132:154104

13. Mathew K, Kolluru V, Mula S, Steinmann SN, Hennig RG. Implicit self-consistent electrolyte model in plane-wave density-functional theory. *J Chem Phys* 2019;151:234101

14. Mathew K, Sundararaman R, Letchworth-Weaver K, Arias T, Hennig RG. Implicit solvation model for density-functional study of nanocrystal surfaces and reaction pathways. *J Chem Phys* 2014;140:084106

15. Wang V, Xu N, Liu J-C, Tang G, Geng W-T. VASPKIT: A user-friendly interface facilitating high-throughput computing and analysis using VASP code. *Comput Phys Commun* 2021;267:108033.

16. Teplensky MH, Fantham M, Li P, et al. Temperature treatment of highly porous zirconium-containing metal-organic frameworks extends drug delivery release. *J Am Chem Soc* 2017;139:7522-7532.

17. Zhang Z, Peh SB, Krishna R, et al. Optimal pore chemistry in an ultramicroporous metal-organic framework for benchmark inverse CO_2/C_2H_2 separation. *Angew Chem Int Ed* 2021;60:17198-17204.

18. Cichocka MO, Liang Z, Feng D, et al. A porphyrinic zirconium metal-organic framework for oxygen reduction reaction: tailoring the spacing between active-sites through chain-based inorganic building units. *J Am Chem Soc* 2020;142:15386-15395.

19. Ma J, Wong-Foy AG, Matzger AJ. The role of modulators in controlling layer spacings in a tritopic linker based zirconium 2D microporous coordination polymer. *Inorg Chem* 2015;54:4591-4593.

20. Feng D, Wang K, Su J, et al. A highly stable zeotype mesoporous zirconium metal-organic framework with ultralarge pores. *Angew Chem Int Ed* 2015;54:149-154.

21. Furukawa H, Gándara F, Zhang Y-B, et al. Water adsorption in porous metal-organic frameworks and related materials. *J Am Chem Soc* 2014;136:4369-4381.

22. Liu TF, Vermeulen NA, Howarth AJ, et al. Adding to the arsenal of zirconium-based metal-organic frameworks: the topology as a platform for solvent-assisted metal incorporation. *Eur J Inorg Chem* 2016;2016:4349-4352.

23. Wang B, Lv X-L, Feng D, et al. Highly stable Zr (IV)-based metal-organic frameworks for the detection and removal of antibiotics and organic explosives in water. *Eur J Inorg Chem* 2016;138:6204-6216.

24. Li Z, Yao B, Cheng C, et al. Versatile structural engineering of metal-organic frameworks enabling switchable catalytic selectivity. *Adv Mater* 2024;36:2308427.

25. Tian S, Wang Z, Gong W, et al. Temperature-controlled selectivity of hydrogenation and hydrodeoxygenation in the conversion of biomass molecule by the Ru_1/mpg-C_3N_4 catalyst. *J Am Chem Soc* 2018;140:11161-11164.

26. Duan H, Liu J-C, Xu M, et al. Molecular nitrogen promotes catalytic hydrodeoxygenation. *Nat Catal* 2019;2:1078-1087.
